# Simulating beach and dune evolution at decadal to centennial scale under rising sea levels

**DOI:** 10.1371/journal.pone.0215651

**Published:** 2019-04-19

**Authors:** Caroline Hallin, Magnus Larson, Hans Hanson

**Affiliations:** Department of Water Resources Engineering, Lund University, Lund, Sweden; Universidade de Aveiro, PORTUGAL

## Abstract

A numerical model for simulating beach-dune evolution at decadal to centennial time scales is developed. The work builds on an existing semi-empirical cross-shore model, the CS-model, to which the effect of sea level rise is added and the routines for aeolian transport and morphological dune evolution are improved. The model development is based on established conceptual models from the literature, which are translated into mathematical formulations and solved numerically. The capability of the proposed model is demonstrated through a case study at Ängelholm Beach, Sweden. The model is calibrated and validated against a seven-year long data set on morphological evolution and sediment grain-size samples. Beach and dune evolution is then simulated from 2018 to 2100 for a range of sea level rise scenarios. The model results are promising, and suggest that the model has potential to be used for long-term assessment of climate change impact on beaches and dunes.

## Introduction

Coastal dunes play an important role to protect against flooding and beach erosion, while providing nature and recreation values [1,2]. Therefore, both natural and artificial dunes are becoming increasingly popular flood protection methods in many coastal areas [3,4]. Meanwhile, rising sea levels increase the probability of flooding as well as the pressure on beaches and dunes [5,6]. The capability to predict future beach and dune evolution is thus of major importance for flood risk assessment and design of coastal protection. Several models have been developed to separately estimate dune erosion during storms [7–9], dune build-up due to aeolian transport [10–12], and long-term coastline evolution [13]. However, models for the evolution of the beach and dune system at decadal time scales, which are of particular interest in coastal management [14], require a coupling between constructive and destructive nearshore, beach, and dune processes [15]. In recent years, there has been advances in the coupling of cross-shore and longshore transport processes at decadal time scales, *e*.*g*., the CoSMoS-COAST model [16] and the LX-model [17]; but, these models do not include dune processes. To improve the understanding of how processes in the land-sea interface control the foredune evolution, Cohn *et al* [18] combined three process-based models—X-BEACH [8], CDM [19], and Aeolis [20]—in the model framework Windsurf. Windsurf successfully simulated dune evolution in one cross-shore transect during one year, however, longer time and spatial scales require reduced complexity approaches. In this study, a model is developed that simulates sediment transport and morphological evolution of the beach and dune on time scales of decades to centuries. The aim is to create a robust, computationally efficient model that couples nearshore, beach, and dune processes.

Simulations of long-term beach and dune evolution require integration of sediment transport processes acting in both the cross-shore and longshore direction on multiple time scales [14,21]. This work builds on the CS-model [15], a semi-empirical beach profile evolution model that simulates sediment transport due to dune erosion and dune overwash caused by wave action, dune build-up from aeolian transport, and beach-bar material exchange. Longshore sediment transport gradients are accounted for as constant sources or sinks, and nourishment episodes can be added to the beach or dune [22]. Previously, sea level rise (SLR) has not been included in the CS-model and the dune build-up from wind-blown sand did not take into account sediment availability, which is an important factor for long-term dune evolution [23,24]. In this study, the CS-model is developed through introducing SLR and mathematical methods to compute aeolian transport and the associated dune growth.

**Fig 1:**
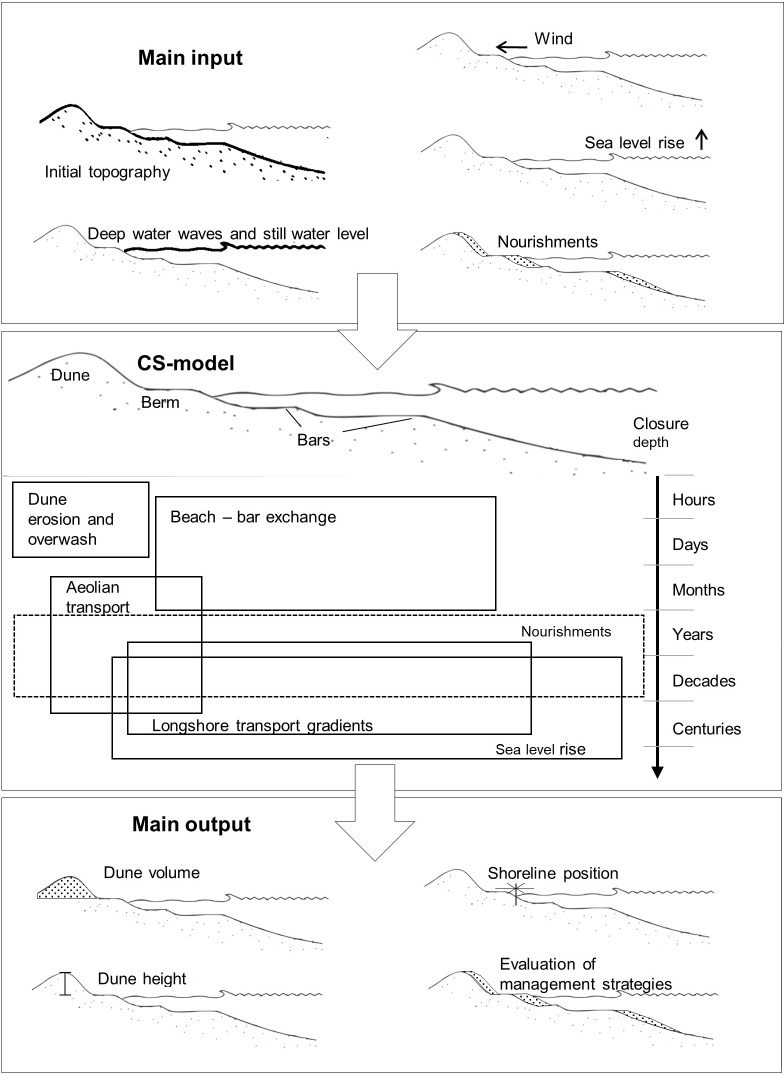
Conceptual model of decadal to centennial scale beach and dune evolution.

The included transport processes are active over a range of spatial and temporal scales (Fig 1). Sediment transport and morphological evolution are simulated in cross-shore transects, which may be aggregated to represent coastal evolution at spatial scales of kilometres. Model input is SLR, wind, deep water waves, and simultaneous still water levels (SWL). From a coastal management perspective model output of interest is *e*.*g*. long-term shoreline change, the shoreline variation envelope, and dune height and dune volume in relation to storm erosion rates [25]. The model is computationally efficient due to reduced complexity transport equations and a simplified representation of the coastal morphology, which makes it suitable for probabilistic approaches.

The main objective of this study is to develop a simple, robust, and computationally efficient model for evolution of the beach-dune system under rising sea levels at decadal to centennial time scales. Established conceptual morphological models are translated into mathematical formulations to extend the CS-model [15] to include the dynamic and interconnected effects of SLR and aeolian transport.

The paper starts by presenting the theoretical background for the proposed model development, followed by a model description. The proposed model is then applied to simulate the long-term beach and dune evolution at Ängelholm Beach, Sweden. First, the model is calibrated and validated against a seven-year data set and thereafter, the evolution from 2018–2100 is simulated considering a range of SLR scenarios. The paper ends with a discussion on model performance and suggestions for further development. Notations used throughout the paper are summarized in a list of symbols at the end.

## Theoretical background

### Aeolian sediment transport

The mechanics of sediment transport by wind and the associated morphological evolution of coastal dune systems have been studied extensively [26,27], which has provided physical insights to aeolian transport processes. Still, existing models do not manage to accurately predict transport rates at time scales relevant for coastal management [23,25,28]. Sediment transport formulas only accounting for wind speed and grain size [26,29–33] are commonly overestimating transport rates when compared to field data from beach environments [23,34].

When modelling yearly to decadal sediment transport rates, limits to transport are of interest, rather than time-varying forcing conditions [23]. Such limiting factors are sediment availability [24], beach width and fetch length [35], beach slope [34], surface moisture [35], snow and ice cover [36], vegetation, and crust development on the beach surface [20].

Dune build-up by wind requires sediment with a grain size fine enough to be mobilised by the wind, and coarse enough to be deposited in the foredune, where the wind shear stress decreases due to the effect of vegetation and topography [35]. Grain sizes in aeolian deposits typically range between 0.15–0.30 mm [37], and are often smaller than those found on the beach [38]. Eroding beaches tend to be drained of fine sediment and accreting beaches to be supplied with fine sediment due to the selection of grain size in transport processes, where smaller grains are more likely to be picked up [39]. In this way, the aeolian sediment transport depends on the longshore and cross-shore transport processes, which control the supply of sediment of appropriate grain size.

### Dune morphology

Transport rates alone cannot be used to predict the morphological evolution of foredunes. It is also important where the aeolian sediment is deposited; on the crest, the seaward or landward slopes. The beach sediment budget, which could be positive, negative, or stable, affects both the rate of transport and the morphological evolution [24,25]. According to Psuty’s conceptual model [24], dunes at an accreting beach will grow rapidly and create a prograding dune ridge topography, where a new foredune is formed in front of the existing ones, creating low dunes with mild slopes [40,41]. The dunes will be low because there is not enough time for them to grow in height before a new foredune is built in front of them [42]. At a stable beach, the dune stays in place and grows higher due to scarping and recovery. Eroding beaches may develop in two different ways; if they are slightly eroding, the dune will maintain or even increase its volume, grow higher, and be displaced inland through scarping in combination with aeolian transport and/or overwash. If the beach is eroding rapidly and overwash processes are dominant, the dune will be flattened and move landward.

**Fig 2:**
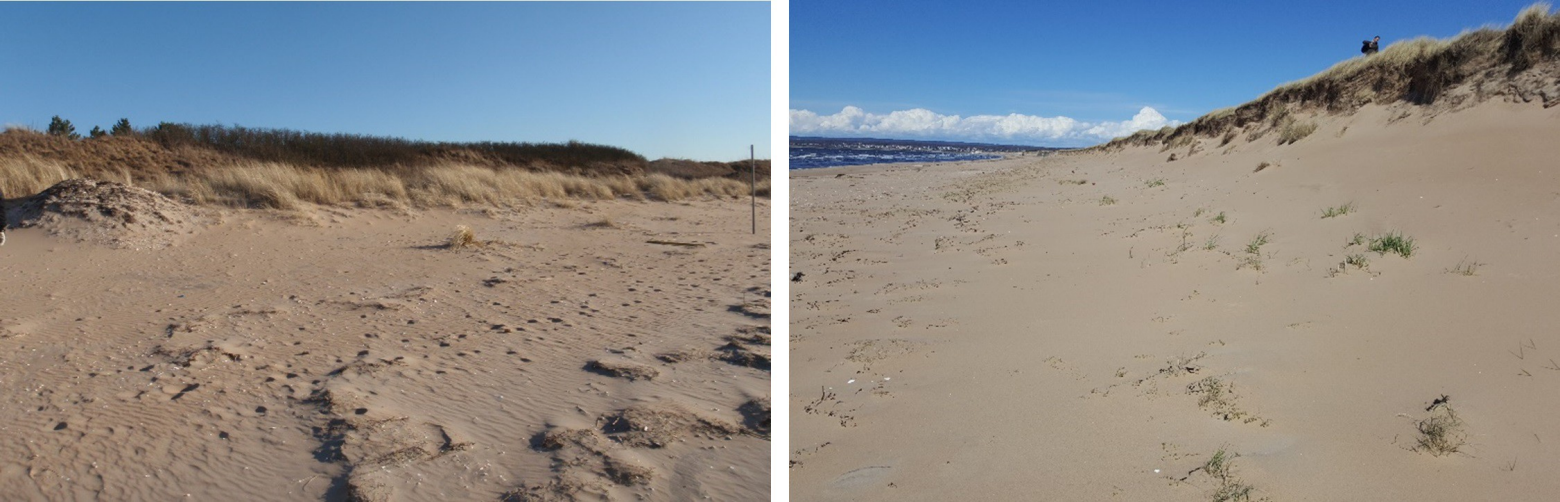
Vegetated embryonal foredune (left) at an accreting beach, and non-vegetated dune ramp (right) at a stable beach in Ängelholm, Sweden. The sites are later referred to as profile C and B, respectively.

The explanatory mechanisms behind these different morphological behaviours are sediment supply and the influence of vegetation. If vegetation is present at the dune foot, sediment is trapped in an embryonal foredune and without vegetation sediment is deposited near the foot of the foredune, forming a dune ramp (Fig 2), which facilitates the passage of wind-blown sand over the dune crest [43,44].

The interaction between dune ramps and morphological evolution was studied at Skallingen spit in Denmark, which is a retreating coastal feature, subject to overwash and landward foredune migration [44]. Christiansen and Davidson-Arnott [44] found that aeolian transport over the dune crest increased rapidly as the vertical distance from the top of the ramp to the crest became equal to or less than 1 m, and that the wind during wet conditions eroded the ramp to approximately this height.

The dune ramp concept was further developed in a study at Greenwich Dunes on Prince Edward Island in Canada [36]. In 2002–2009 measurements were performed at two stretches of a beach with different littoral sediment budgets; one negative and one positive or neutral, although both showing a long-term transgressive trend. After scarping, if no ramp was present, very little sediment reached the dune crest or landward slope. When a dune ramp was present, accumulation was observed at the upper seaward slope, crest and landward slope. The beach with a temporarily positive sediment transport had vegetated embryonal dunes present for 2–3 years and through that time most sediments were deposited at the seaward side of the dune. When the embryonal dune was damaged and a vegetated ramp had not yet been formed, the observed transport to the crest and landward side of the dune was significant. These studies support that the sediment budget affects the morphological evolution, since embryonal foredunes are more likely to occur on accreting beaches whereas dune ramps form on eroding or stable beaches.

For long-term modelling of foredune evolution, dune height is essential as it affects flood safety. The conceptual models of Psuty [24] and Sherman and Bauer [25] suggest that dune height is depending on the sediment budget. The ratio between height/width of coastal foredunes are typically about 0.11 [12], but the maximum height varies between different environments. Durán and Moore [19] proposed a linear relationship between foredune height and wave height, assuming that the dune height was depending on wave impact. Hesp [27] found a strong relationship between increasing foredune height and increasing dissipativeness but concluded that the trend might also be explained by higher exposure to storm winds at dissipative beaches. In summary, the dune height is site-specific and depends on the sediment budget, wind and wave climate, and sediment and vegetation characteristics.

### The Bruun rule

The Bruun rule provides a simple method to estimate the shoreline retreat, *R*_*Bruun*_, under a slowly rising sea level [45],

$$R_{Bruun}=S_{SLR}\frac{B_{Bruun}}{h}$$ (1)

where *S*_*SLR*_ is the sea level rise, *B*_*Bruun*_ and *h* the width and the height of the active profile, respectively. The width *B*_*Bruun*_ represents the distance from the shoreline to the depth closure, and *h* is the sum of the depth of closure and the beach berm height.

The Bruun rule is based on the assumption that SLR creates accommodation space for sediment within the subaqueous part of the active profile. The Bruun Rule only considers cross-shore transport, assuming that the profile consists entirely of sand, that there is a full spectrum of waves, wind and water levels to force the profile to its new equilibrium, and that the SLR is slow. All sediment transport is in the seaward direction to the subaqueous part of the profile. However, infilling sediment can come from various sources, not only the beach. The sediment may also originate from a negative longshore sediment transport gradient, artificial nourishments, or from offshore supplies outside the depth of closure, also known as the Dean equilibrium concept [46–48].

The original version of the Bruun rule does not consider any vertical adjustment of the subaerial part of the profile to SLR. If the mean sea level rises, also the beach berm height and dune foot are expected to adjust to the new sea level [49]. For this purpose, extended versions of the Bruun Rule that accounts for landward transport due to aeolian processes and overwash of the beach berm and dune have been proposed [49–51].

It should be noted that the Bruun rule is a much discussed method that has been subject to numerous critical evaluations [52]. The first main argument against the Bruun rule concerns its two-dimensionality and the assumption that other transport processes such as gradients in longshore transport and overwash are excluded. In the CS-model, however, the Bruun rule will only be applied to compute the transport directly related to SLR. Other relevant transport processes are dealt with in separate transport equations, and the morphological evolution of the beach and dune is a result of all of these types of transports.

The second common critique against the Bruun rule is the validity of the concepts of equilibrium profiles and depth of closure. In a recent extensive laboratory study [53], both the original Bruun rule [45] and a modified version including landward transport [49] showed good agreement with observations and confirmed the underlying assumption of a profile adjustment to SLR.

Last, the Bruun rule has also been criticized for ignoring import of sediment from deeper water outside depth of closure [47]. However, these processes are more common at geological time scales than at engineering time scales. If known to be present they could be added as a sediment source in the model.

## Model for long-term foredune and beach evolution

The CS-model simulates sediment transport and the associated morphological evolution based on a set of transport and continuity equations [15]. In this paper, the profile schematization is developed and routines are added for aeolian transport, morphological dune evolution, and the effect of SLR. For more detailed description of the other model components we refer to Larson *et al* [15].

### Profile schematization

**Fig 3:**
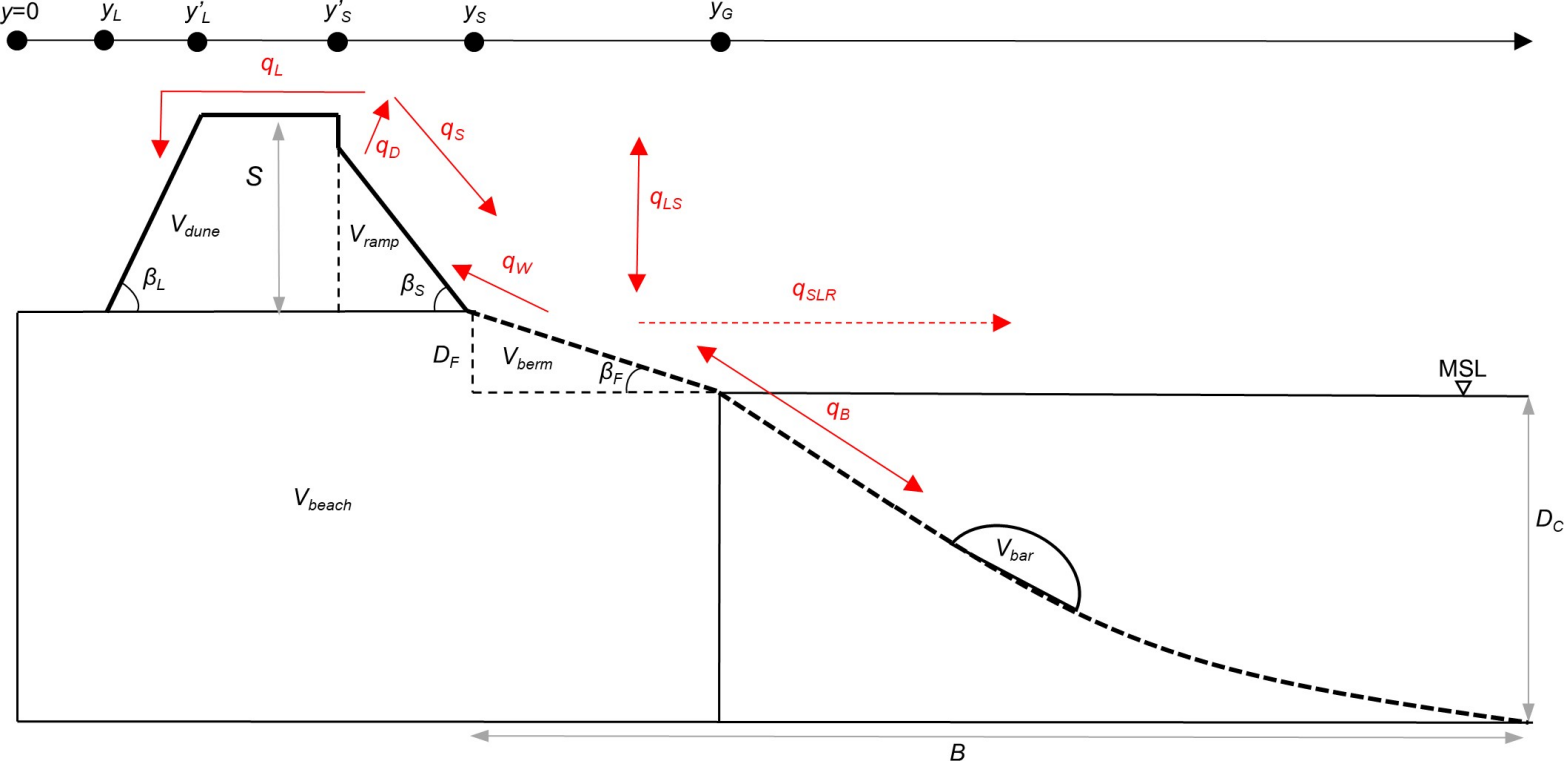
Definition of heights, length coordinates, volumes and transports (red arrows).

The beach profile is schematized through distances, slopes, characteristic heights, and volumes (Fig 3), which are updated every time step. Length coordinates, relative to a reference point behind the dune, are defined for landward and seaward dune foot, *y*_*L*_ and *y*_*S*_, and dune crest, *y’*_*L*_ and *y’*_*S*_, and the intersection with mean sea level, *y*_*G*_. Landward and seaward dune slopes, tan *β*_*L*_ and tan *β*_*S*_, are assumed to be constant and a maximum foreshore slope, tan *β*_*F*,*max*_, is defined. If tan *β*_*F*,*max*_ is exceeded, *β*_*F*_ *>β*_*F*,*max*_, the entire beach is considered a swash zone. Under this condition, sediment that would have been eroded from the beach is instead eroded from the dune.

Depth of closure, *D*_*C*_, and dune foot height, *D*_*F*_, are constants referenced to the mean sea level (MSL). The dune height, *S*, is a variable, specified relative to *D*_*F*_. A maximum value of *S*, *S*_*max*_, is limiting dune height growth if the sediment budget is positive, whereas for negative and stable budgets *S* has no restrictions [12]. *S*_*max*_ can be approximated as a representative height of prograding dune ridges, present in or near the study area.

Volumes and transport rates are given per meter of beach width. The specified volumes are dune volume, *V*_*dune*_, where the dune ramp volume, *V*_*ramp*_, is an integral part, total beach volume, *V*_*beach*_, where the berm volume, *V*_*berm*_, is an integral part, and bar volume, *V*_*bar*_ (Fig 3). The bar volume represents nearshore subaqueous deposits involved in material exchange with the beach and may consist of none, one, or multiple bars. The rest of the subaqueous part of the profile, from MSL to *D*_*C*_, is assumed to be in equilibrium [54].

The beach volume *V*_*beach*_ is defined as the volume of sediment from a reference point behind the dune to the intersection with mean sea level, *y*_*G*_, which is vertically limited by the depth of closure, *D*_*C*_, and the dune foot height, *D*_*F*_. The sub-volume *V*_*berm*_ is used to calculate beach width; the quantity *y*_*G*_*-y*_*S*_ is defined as a function of *V*_*berm*_ based on site specific data. Thus, the shape of the beach is not specified; it is only described by its width and volume, indicated with a dashed line in Fig 3.

The purpose of the ramp volume *V*_*ramp*_ is to determine how the sediment distribution over the dune occurs. If the dune is eroded, *V*_*ramp*_ is eroded first and if the ramp disappears completely, the main part of the dune (behind the seaward dune crest, *y’*_*S*_) will start to erode.

The changes in profile schematization compared to previous versions of the CS-model require adjustment of the frictional losses over the beach. The runup height significant for dune impact, *R*, is defined as $R=0.158\sqrt{H_{rms}L_{0}}$, where *H*_*rms*_ and *L*_*0*_ are deep water root-mean-square wave height and mean wavelength, respectively [55]. *R* is corrected for friction over the beach according to [56],

$$R'=R\exp(-2c_{f}x)+(D_{F}-SWL)(1-\exp(-2c_{f}x))$$ (2)

where *R’* is the adjusted runup height, *c*_*f*_ is an empirical friction coefficient, and *x* is the horizontal travel distance of the wave front. If *SWL*+ *R’* > *D*_*F*_ dune erosion will occur. Since the beach in the CS-model has no shape, the travel distance cannot be exactly known. Instead the travel distance *x* is expressed as a function of the volume *V*_*berm*_,

$$x=\frac{2V_{berm}}{D_{F}}\left(1-\frac{SWL}{D_{F}}\right)$$ (3)

### Aeolian sediment transport

The proposed aeolian sediment transport scheme is based on the assumption that, on a decadal time scale, the most important limiting factor for aeolian sediment transport is the supply of material of appropriate grain size. Sediment available for aeolian transport is assumed to be present as a location-specific fraction of the transported sediment volumes longshore and cross-shore. Availability is computed through a balance including sediment transport and nourishments, to and from *V*_*beach*_. The volume of sediment available for aeolian transport, *V*_*W*_, in time step *i* is calculated as,

$$\begin{array}{l} V_{W,i}=V_{W,i-1}+(q_{S,i-1}-q_{W,i-1}-A{\;}_{q}\;(q_{SLR,i-1}-q_{LS,i-1}))\Delta t+A_{b}V_{nour,i-1}\\ V_{W}\geq 0 \end{array}$$ (4)

where *q*_*S*_ is the transport rate of eroded sediment from the dune to the beach, *q*_*W*_ is the aeolian transport rate from the beach to the dune, *q*_*SLR*_ is the transport rate of sediment to compensate for SLR, ‘Bruun Rule’ transport, *q*_*LS*_ is the transport rate due to gradients in longshore transport, *Δt* is length of time step, and *V*_*nour*_ volume of beach nourishments. The coefficients *A*_*q*_ and *A*_*b*_ describe the fractions of the transport rate and nourished volume, respectively, that are within the proper range of grain size and available for aeolian transport.

If *V*_*W*,*i*_ > 0, aeolian transport will take place during time step *t* = *i*. If *V*_*W*,*i*_ ≤ 0, the aeolian transport will be turned off and *V*_*w*,*i*_ is set to zero. The initial available volume, *V*_*w*,*0*_, depends on conditions prior to the simulation period, *e*.*g*. nourishments and large dune erosion events.

The potential aeolian sediment transport rate, *m*_*WE*_, in kg/s/m, can be estimated by [31],

$$m_{WE}=K_{W}\sqrt{\frac{D_{50}}{D_{50}^{ref}}}\rho_{a}\frac{u_{*}^{2}}{g}\left(u_{*}-u_{*c}\right)$$ (5)

where *D*_*50*_ is the median grain size, *D*_*50*_^*ref*^ is the median reference grain size of 0.25 mm, ρ_a_ the density of air, *g* the acceleration due to gravity, u_*_ the shear velocity at the bed, u_*c_ the critical shear velocity at the bed, and *K*_*w*_ an empirical coefficient. If u_*_ < u_*c_, then *m*_*WE*_ = 0.

The shear velocity, u_*_, can be calculated using the law of the wall,

$$\frac{u_{z}}{u_{*}}=\frac{1}{\kappa }\ln\left(\frac{z}{z_{0}}\right)$$ (6)

where *u*_*z*_ is the wind velocity at *z* meter above ground, *z*_*0*_ is the aerodynamic roughness height, and *κ* is von Karman’s constant (≈ 0.41). For wind conditions below the critical shear velocity for initiation of transport, u_*_<u_*c_, the roughness height, *z*_*0*_, can be parameterized as *z*_*0*_ = 0.081log_10_(*d*/0.18), where *d* is the grain size in mm [33]. The critical shear velocity can be estimated through [26],

$$u_{*c}=A_{W}\sqrt{\frac{\left(\rho_{s}-\rho_{a}\right)}{\rho_{a}}gD_{50}}$$ (7)

where ρ_*s*_ is the density of sand (typically 2650 kg/m^3^) and *A*_*W*_ is a coefficient of about 0.1.

To determine the shear velocity during transport, two wind measurements at different heights or one wind measurement and an estimate of the roughness height affected by transport are required. For long-term simulations, wind data is typically not available from the beach of interest but from a wind gauge that is not influenced by the sand transport. Considering the spatial and temporal resolution of available wind data, the relationship between *u*_*z*_ and u_*_ can be approximated as linear [57] so that *m*_*WE*_∝*u*_*z*_^2^(*u*_*z*_-*u*_*z*,*c*_) [58]. The critical velocity for transport at *z* m height, *u*_*z*,*c*_ can be computed through combining Eqs 6 and 7. Eq 5 can then be rewritten as,

$$m_{WE}=C_{W}\sqrt{\frac{D_{50}}{D_{50}^{ref}}}\rho_{a}\frac{u_{z}^{2}}{g}\left(u_{z}-u_{z,c}\right)$$ (8)

where *C*_*W*_ is an empirical coefficient.

The mass flux *m*_*WE*_ is converted to a volumetric equilibrium transport rate (*q*_*WE*_) of sand to the dunes by,

$$q_{WE}=\frac{m_{WE}}{\rho_{s}(1-P)}$$ (9)

where *P* is the porosity (typically 40%).

To reach the transport rates described by the equilibrium equations, a critical fetch length is required [35,59]. The fetch length, *F*, depends on the wind angle towards shore normal, θ, and the dry beach width, *B*_*dry*_. For 0° ≤ θ ≤ 80°,

$$F=\frac{B_{dry}}{\cos(\theta )}$$ (10)

The beach is assumed to be dry landward of the runup limit. A runup length coordinate, *y*_*R*_, is defined as,

$$y_{R}=y_{S}+\left(1-\frac{R+SWL}{D_{F}}\right)\left(y_{G}-y_{S}\right)$$ (11)

The dry beach width, *B*_*dry*_, is then calculated as the horizontal distance from the runup limit to the seaward dune foot *B*_*dry*_ = *y*_*R*_-*y*_*S*_. If *y*_*R*_ ≥ *y*_*S*_, the whole beach is assumed to be wet and there will be no aeolian transport.

A simplified equation for the potential transport rate, corrected for fetch-limited conditions, *q*_*WF*_, was developed by Larson *et al* [15] based on the work by Sauermann *et al* [60],

$$q_{WF}=q_{WE}\left(1-\exp\left(-\delta \;F\right)\right)$$ (12)

where δ is an empirical coefficient in the order of 0.1–0.2 m^-1^.

Oblique wind angles have longer fetches and may therefore generate higher aeolian transport rates. This effect is counteracted by the cosine effect, implying that only the onshore component, *q*_*W*_, adds to the dune volume,

$$q_{W}=q_{WF}\cos(\theta \;)$$ (13)

Aeolian transport due to offshore directed wind is neglected.

When neglecting the impact of grain size, the effect of slope correction on transport rates has been found to be small at beaches with slopes below 15° [34]. Here, grain size is accounted for in Eqs 5 and 6 and since most beach slopes are milder than 15° [61], the effect of beach slope is assumed to be negligible.

### Morphological dune evolution

Dunes evolve differently depending on where the aeolian transported sediment is deposited, in front of the dune, on the seaward slope, on the crest, or on the landward slope. Here we introduce a sediment deposition scheme based on Psuty’s sediment budget formulation [24] and the dune ramp concept [36,44].

**Fig 4:**
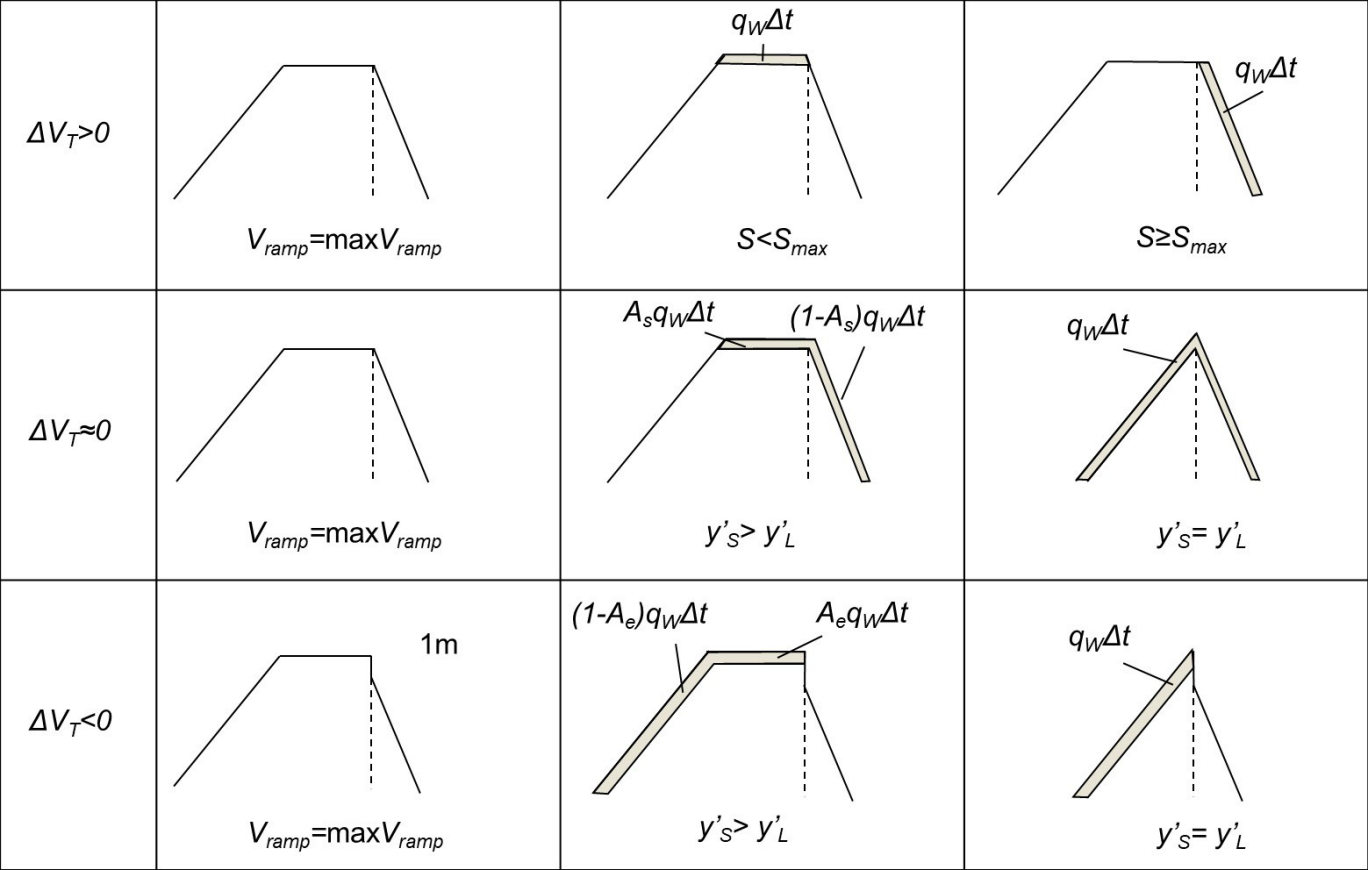
Sediment distribution scheme on a dune due to wind-blown sand.

The sediment budget is calculated as the change of volume in the beach-dune system (*ΔV*_*T*_) over a relevant time scale (*T*_*bud*_) and can either be negative (*ΔV*_*T*_ < 0), stable (*ΔV*_*T*_ ≈ 0), or positive (*ΔV*_*T*_ > 0),

$$\Delta V_{T}=\frac{1}{n}\sum_{t=i-n}^{t=i}\left((-q_{SLR,t}+q_{LS,t})\Delta t+V_{nour,t}\right)$$ (14)

where n = *T*_*bud*_/Δt. Here all nourishments are considered in *V*_*nour*_, also dune nourishments. The time scale, *T*_*bud*_, is in the order of years and should be long enough to represent long-term trends and not seasonal variations. The proposed distribution scheme generates different dune evolution depending on the sediment budget (Fig 4).

In the case of a **positive** sediment budget, *ΔV*_*T*_ > 0, the ramp is filled until the ramp height is equal to the dune height, *S*. After the ramp has filled up, if *S* < *S*_*max*_, for a trapezoidal shape, the sediment will be deposited on the crest, and for a triangular shape, the dune grows while maintaining a constant dune crest length coordinate, *y’*_*L*_ = *y’*_*S*_ = constant, until *S*_*max*_ is reached. If *S* ≥ *S*_*max*_, all sediment is deposited on the seaward side of the dune and the dune grows seaward maintaining a trapezoidal shape.

For a **stable** sediment budget, *ΔV*_*T*_ ≈ 0, the ramp is filled until the ramp height is equal to the dune height, *S*. Thereafter, in the case of a trapezoidal dune shape, a fraction of the sediment, *A*_*s*_, is deposited on the crest and the fraction 1-*A*_*s*_ is deposited on the seaward side of the dune. If the dune has a triangular shape, the dune grows in height while maintaining a constant dune crest length coordinate, *y’*_*L*_ = *y’*_*S*_ = constant.

When the sediment budget is **negative**, *ΔV*_*T*_ < 0, the ramp is filled until a critical ramp height is reached. Thereafter, in the case of a trapezoidal dune shape, a fraction of the sediment, *A*_*e*_, is deposited on the crest and the fraction 1–*A*_*e*_ is deposited on the landward side of the dune. If the dune has a triangular shape, *y’*_*L*_ = *y’*_*S*_, the dune grows on its landward side.

The critical ramp height is here defined as 1 m below the crest level [44]. If the model is applied in an area where local observations suggest otherwise, a site-specific value of the critical ramp height should be adopted. If there are no observations of sediment distribution across the dune, the distribution coefficients, *A*_*s*_ and *A*_*e*_, can be set to 0.5 based on the observations by Ollerhead *et al* [36].

### Impact of sea level rise

**Fig 5:**
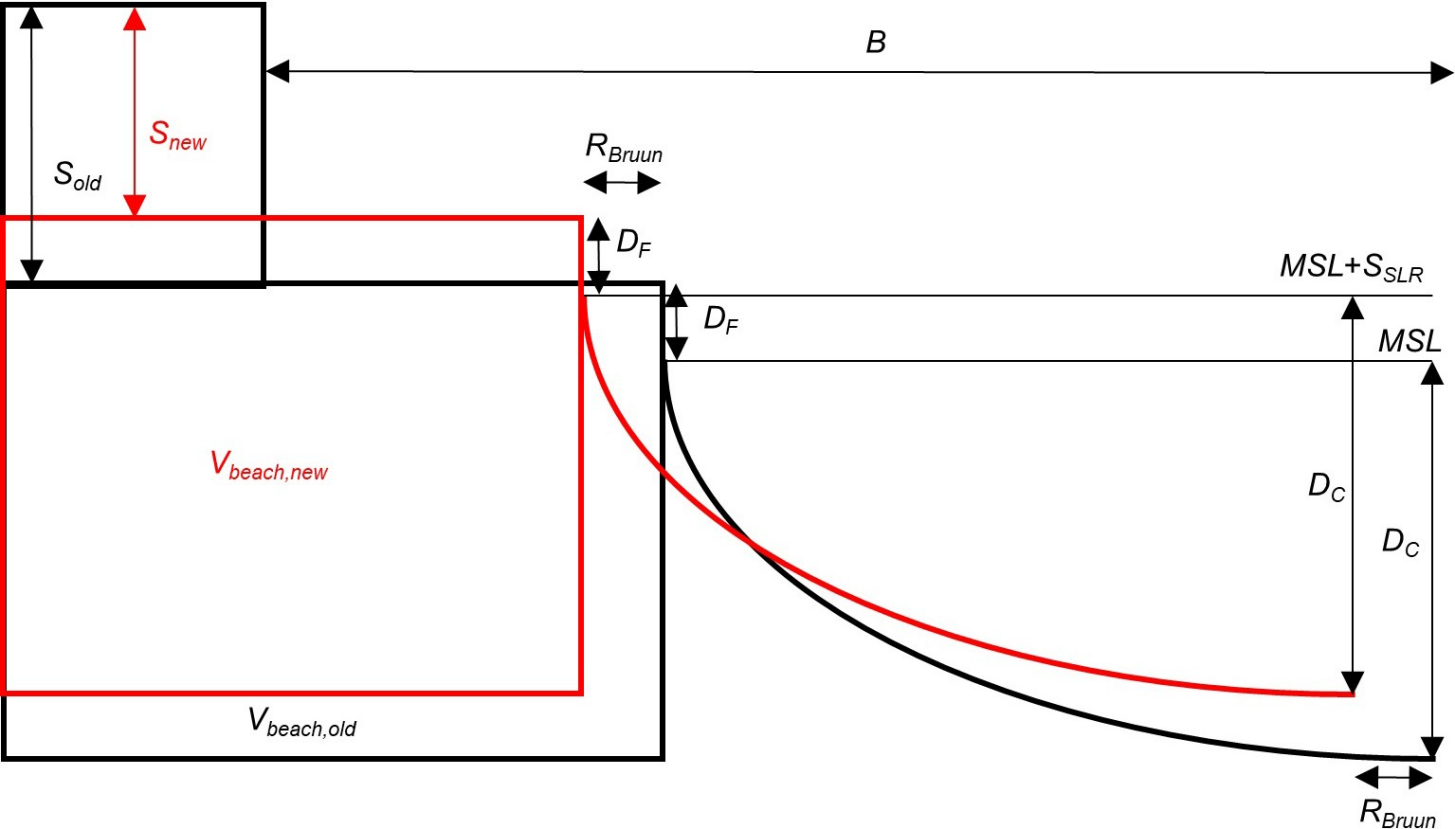
Impact of sea level rise on beach volume and dune height. The beach and dune volumes are for simplicity of the visualisation schematized as rectangular shapes.

The Bruun Rule is used to calculate the required sediment transport from the beach volume, *V*_*beach*_, to the active part of the profile to compensate for SLR. In agreement with previous Bruun rule extensions [49–51], also landward transport is accounted for to compensate for an elevation adjustment of the dry beach (Fig 5). Analogous to the original Bruun Rule concept, the dry beach is assumed to be subject to hydrodynamic forcing capable of adjusting it to a slowly rising sea level. The explanatory mechanism is that the beach is built by waves; with a higher MSL the waves will build a higher beach. Therefore, the width of the active profile, *B*, is here extended to encompass the horizontal distance from the seaward dune foot, *y*_*S*_, to the depth of closure, *D*_*C*_.

The dune foot height is kept constant relative to MSL. Thus, the dune foot is moving up with the same rate as SLR. Since the dune crest elevation is not changing, the lowest part of the dune is per definition converted from dune volume, *V*_*dune*_, to beach volume, *V*_*beach*_. Also the depth of closure, *D*_*C*_, is constant, so that the lower vertical limit of the beach volume is moving up with the same rate as SLR.

*V*_*beach*_ is eroding to supply the active profile with sediment equivalent to the demand from the increased accommodation space due to SLR. The difference between the old and new *V*_*beach*_ is denoted *V*_*SLR*_. Thus, the volume *V*_*SLR*_ eroded from the beach is derived from the Bruun Rule with the new definition of the active profile width, *B*,

$$V_{SLR}=R_{Bruun}(D_{F}+D_{C})=S_{SLR}B$$ (15)

The sediment transport required to maintain the equilibrium profile, *q*_*SLR*_, is taken as the time derivative of *V*_*SLR*_,

$$q_{SLR}=\frac{dV_{SLR}}{dt}=\frac{dS_{SLR}}{dt}B$$ (16)

Consequently, there are two direct effects of SLR in the CS-model: first, adjustment of the equilibrium profile to the new sea level by an offshore ‘Bruun rule’ transport, *q*_*SLR*_, from the beach volume *V*_*beach*_; and second, decrease of dune volume, *V*_*dune*_, and height, *S*, due to an upward shift of the reference SWL (Fig 5). The aeolian transport and morphological dune evolution scheme are indirectly impacted through *q*_*SLR*_ (Eqs 4 and 14). Furthermore, the ‘Bruun rule’ transport is a sink for the beach volume, which will affect wave energy dissipation over the beach (Eqs 2 and 3). This leads to an increase in the probability of dune erosion and overwash transport, which are processes already included in the CS-model [15]. In this way, the dune may translate landwards and the new scheme for morphological dune evolution allows the dunes to adjust in height to the new sea level by keeping *S*_*max*_ constant relative the dune foot elevation.

## Case study Ängelholm Beach, Sweden

**Fig 6:**
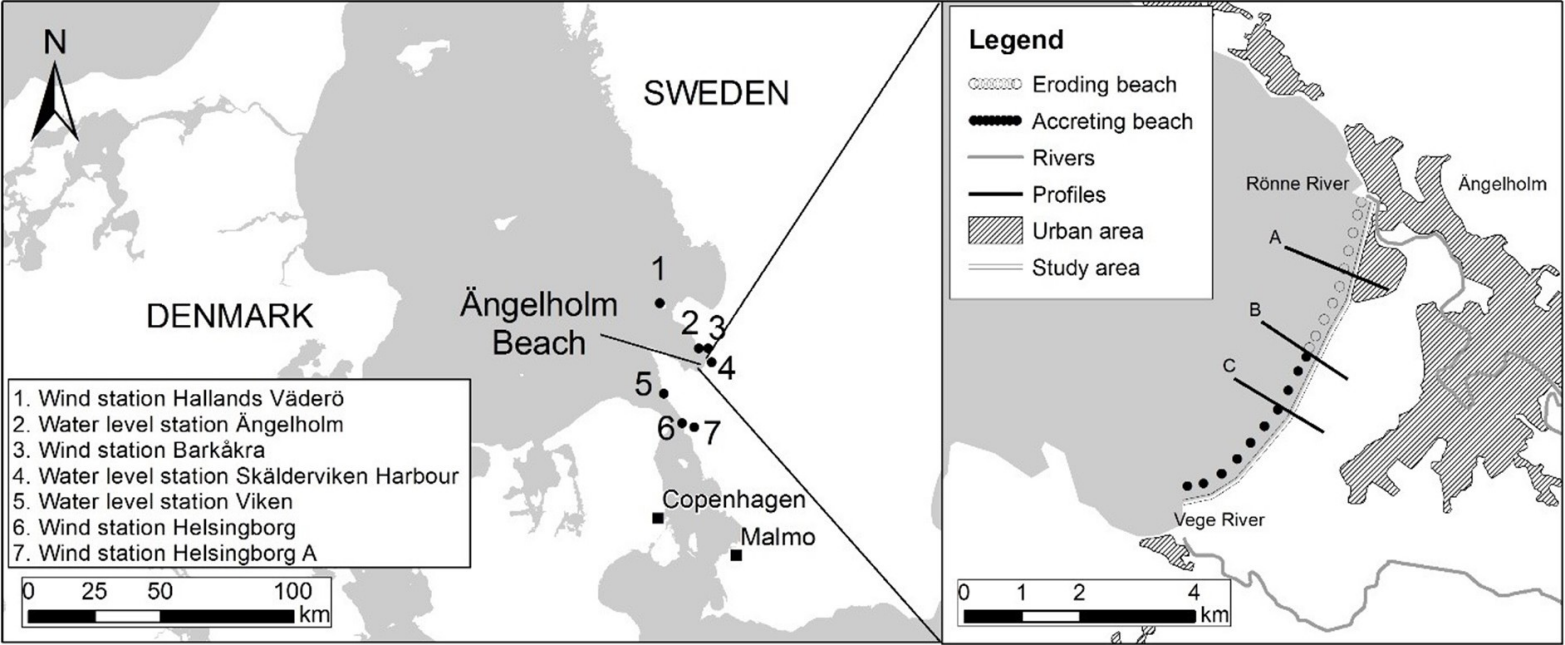
Maps over the study area and measurement stations.

To demonstrate the capability of the proposed model to simulate long-term beach and dune evolution, it was applied to a seven-year data set from Ängelholm Beach in south Sweden (Fig 6).

### Forcing

**Fig 7:**
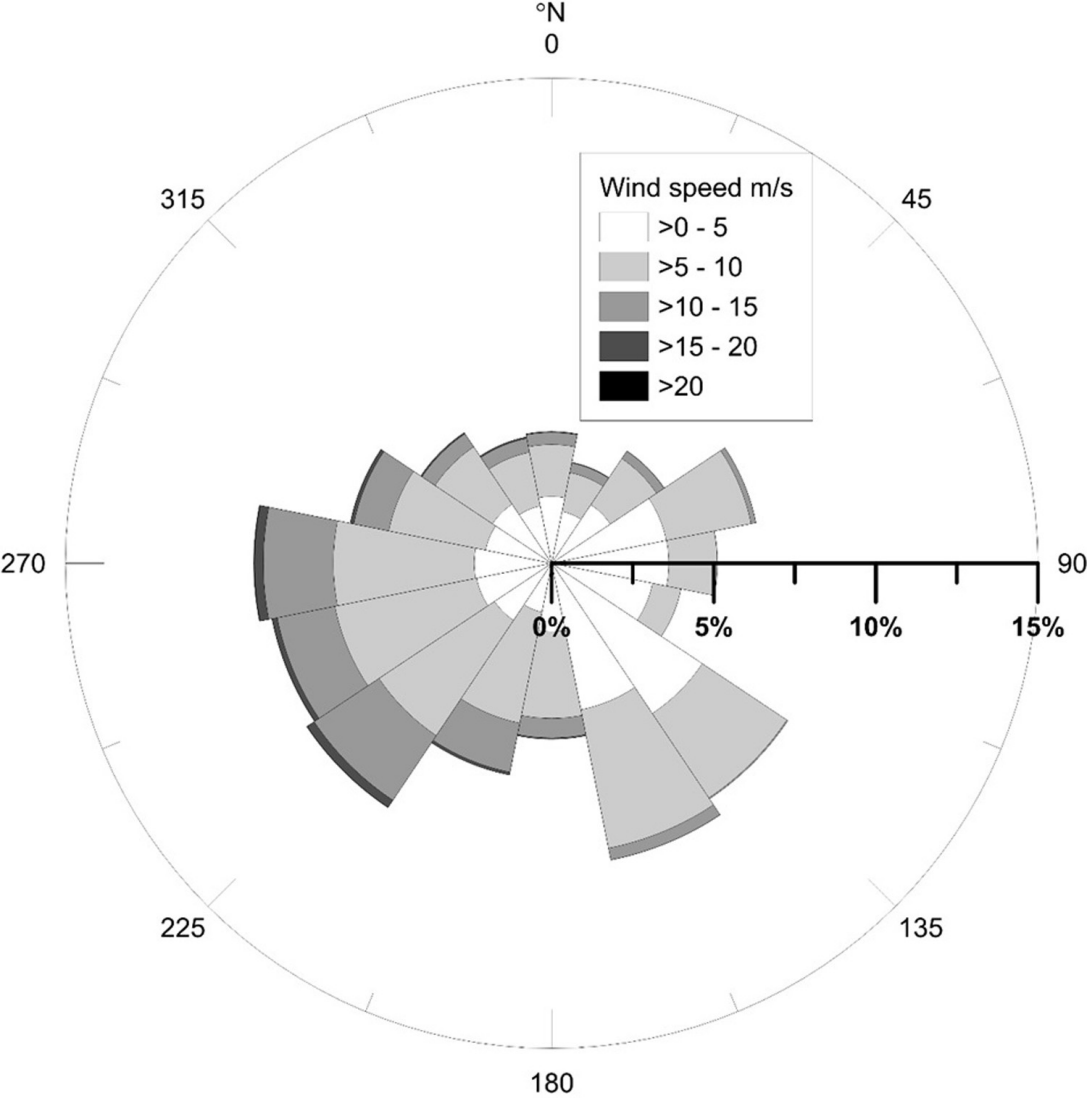
Wind rose compiled with data from 1976–2017.

Wind data at 10 m height was collected from stations 1, 3, 6, and 7 (Fig 6), operated by the Swedish Meteorological and Hydrological Institute (SMHI). Predominant wind direction is west to southwest and the shore normal is oriented 310 ⁰N (Fig 7).

**Fig 8:**
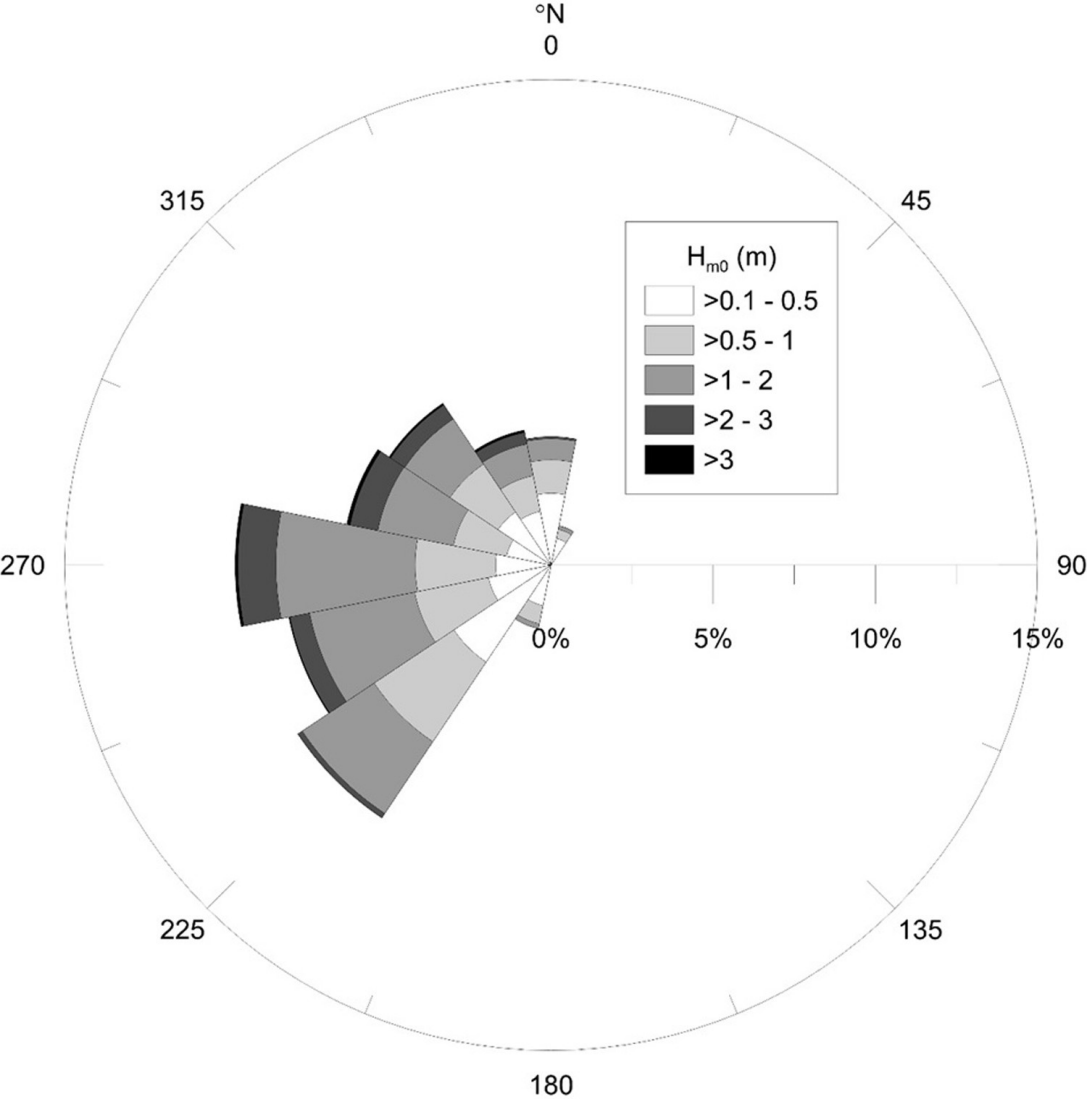
Hindcasted significant wave height at the mouth of Skälderviken Bay during 1976–2017.

Deep-water wave conditions at the mouth of Skälderviken Bay were hindcasted from the wind data using a modified SMB-method [22,62] (Fig 8). The largest computed significant wave height and peak period is 5.3 m and 9.3 s, respectively, which occurred on 06/12/2013.

**Fig 9:**
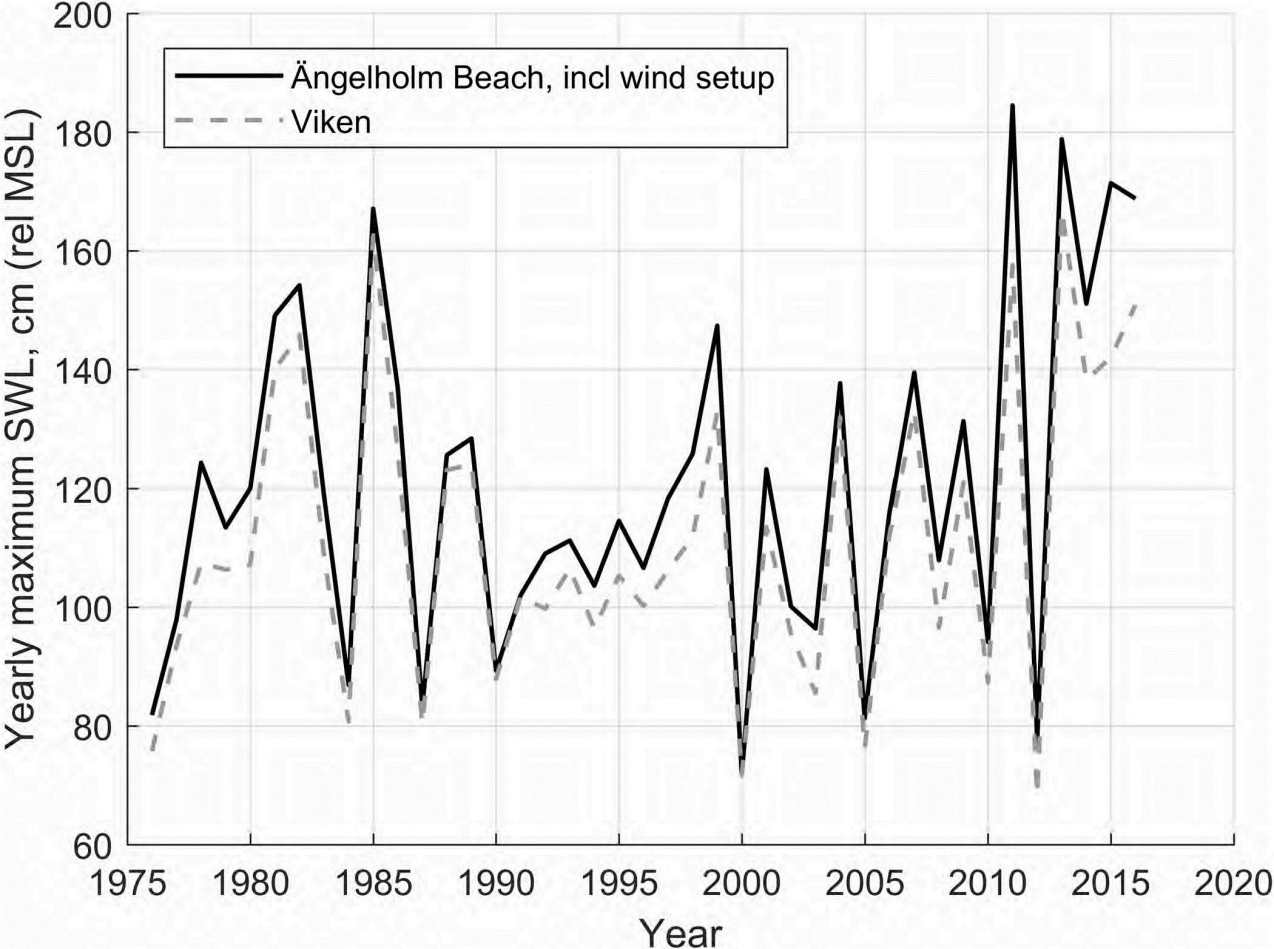
Yearly maximum *SWL* in 1976–2016 based on measurements from Viken (dashed grey line) and the yearly maximum corrected for local wind setup in Skälderviken Bay (solid black line).

Water level data was collected from SMHI station 5 (Fig 6). The water levels were corrected for local wind setup, following the method described by Palalane *et al* [22]. The current decade has seen several extreme events compared to the period 1990–2010 (Fig 9). The highest observed water level, adjusted for wind setup, was 185 cm above MSL on 27/11/2011. There is no significant astronomical tide.

### Morphology

**Fig 10:**
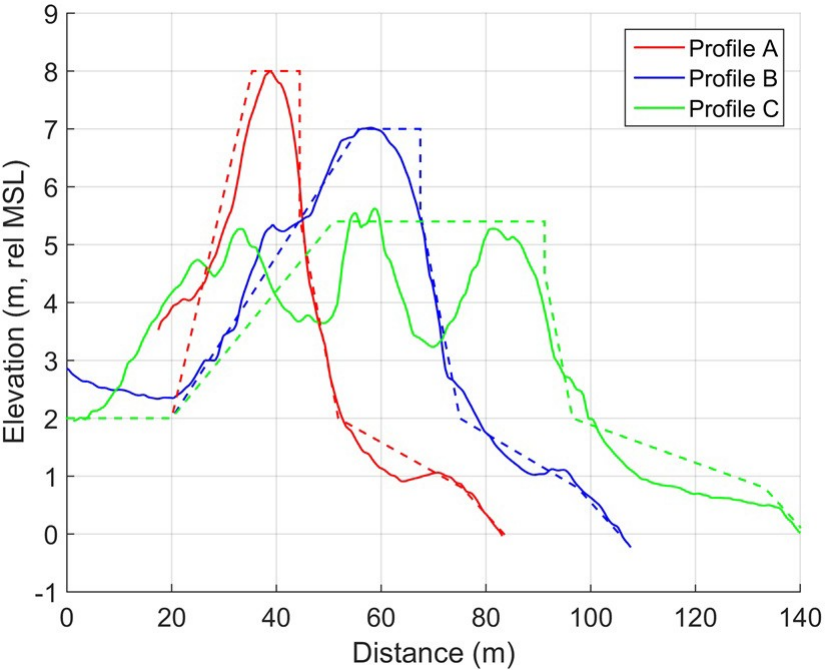
Profiles from DEM dated 12/04/2010, dashed lines display model schematization of the dune.

Beach morphology varies alongshore, with higher dunes and a narrower beach in the north, and lower dunes and a wider beach in the south. The observed long-term coastline evolution is erosion in the north and accretion in the south (Fig 6). Profiles A, B, and C have been selected to represent stretches of the beach with different long-term evolution (Fig 10). Analysis of aerial photos since the 1940’s suggests that the vegetation line has been retreating about 0.3 m/year in profile A, stable in profile B, and accreting about 0.3 m/year in profile C [22].

Topographic observations are available from 2010–2017. In Table 1, the dune volume changes over time are presented together with the data source.

**Table 1:** Dune volume changes between consecutive measurements. A summation of the changes since the first topographic observation in 2010 is given within brackets.

	Volumetric change since previous survey in m^3^/m *(accumulated change since 2010)*			
Survey date	Profile A	Profile B	Profile C	Data source
12/04/2010	initial profile	initial profile	initial profile	DEM LiDAR (resolution 1×1 m)
30/11/2012	-2 *(-2)*	-1 *(-1)*	1 *(1)*	DEM LiDAR (resolution 1×1 m)
26/11/2014	13 *(11)*	-10 *(-11)*	-10 *(-9)*	DEM Photogrammetry (resolution 1×1 m)
21/01/2015	-28 *(-17)*	-1 *(-12)*	-2 *(-11)*	DEM Photogrammetry (resolution 1×1 m)
30/09/2015	13 *(-4)*	0 *(-12)*	2 *(-9)*	Profile RTK-GPS
15/12/2015	-14 *(-18)*	-2 *(-14)*	-2 *(-11)*	DEM Photogrammetry (resolution 1×1 m)
22/04/2016	3 *(-15)*	-1 *(-15)*	0 *(-11)*	Profile RTK-GPS
27/10/2016	0 *(-15)*	0 *(-15)*	0 *(-11)*	Profile RTK-GPS
05/01/2017(A,C) 17/01/2017(B)	-10 *(-25)*	1 *(-14)*	-1 *(-12)*	Profile RTK-GPS

Storms causing dune erosion occurred in November 2011, December 2013, January 2015, December 2015, and December 2016, of which the storms in 2011 and 2013 were the most severe.

Since 2010, the dune volume has decreased in all three profiles, including the long-term stable and accreting profiles, B and C. This is mainly due to the storm erosion in December 2013 from which the dunes have not recovered. In profile A, the observed accretion in November 2014 and September 2015 is due to dune nourishments when sediment was scraped from just seaward of the water line and deposited in the dune front. These operations were carried out in April 2012, April 2014, and March 2015 to replace the sediment lost from the dunes during storms.

In a bathymetric survey from October 2012 (provided by the Swedish Geological Survey) no bars are observed in the subaqueous part of the studied profiles. However, from aerial photos, parallel bars can be detected south of profile C. Along the entire beach temporary crescentic subaqueous deposit are visible within 70 m from the shoreline. These features indicate that there is a cross-shore exchange of sediment stored in nearshore deposits, although, no fully developed bars are observed within the measured profiles.

### Sediments

**Fig 11:**
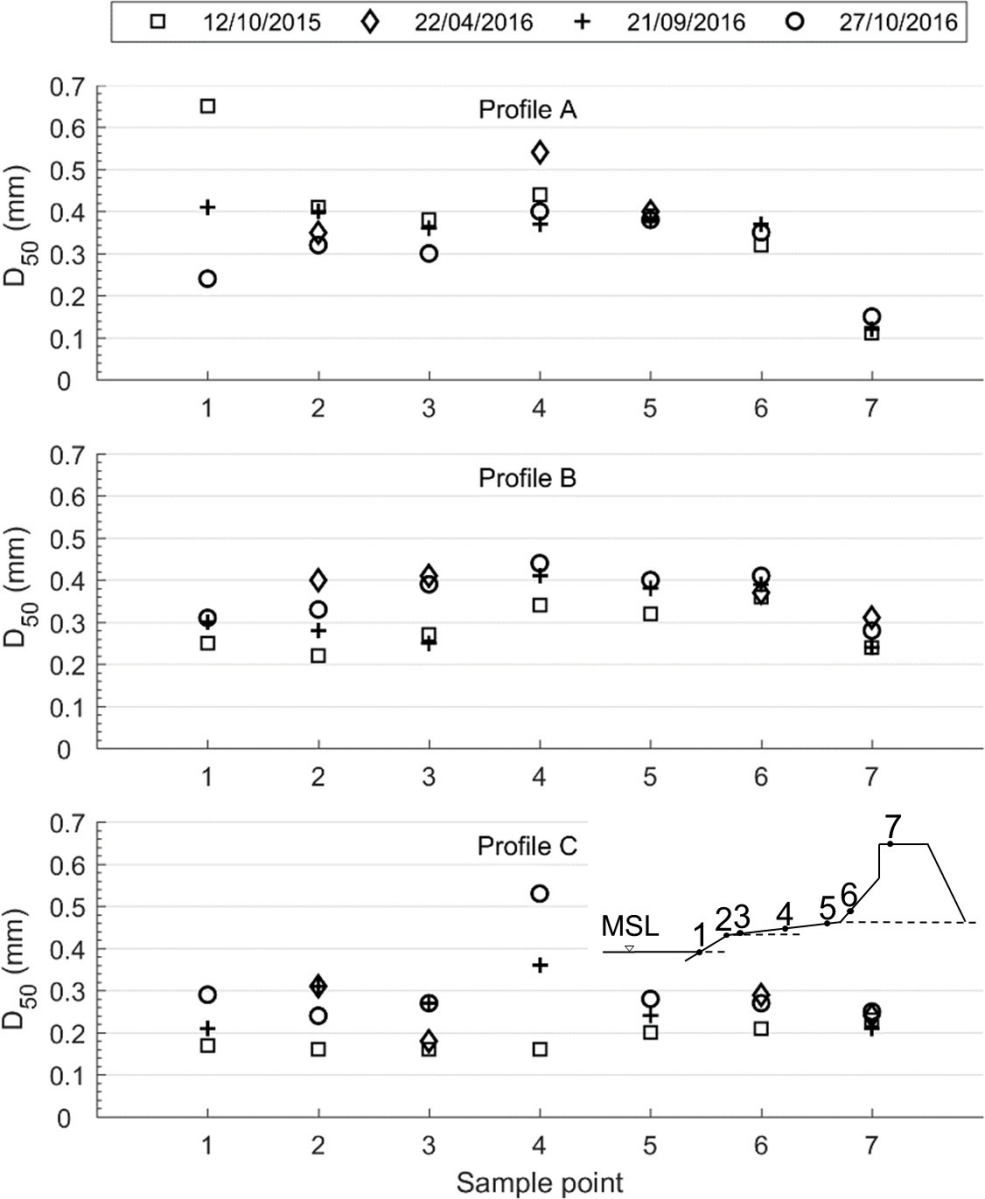
Median grain size D_50_ for sediment samples in Profile A, B, and C sampled from the shoreline (1) to the dune crest (7).

Sediment samples have been collected at four occasions during 2015 and 2016 to determine *D*_*50*_ across the beach and dune (Fig 11).

The median grain size at sample points 1–5 on the foreshore and berm is in general decreasing from profile A to profile C, indicating a longshore sorting. As the sediment is fairly well graded, the grain size found in the dune, about 0.2–0.3 mm, is assumed to be representative for dune building sediments. The samples from the dune in profile A with lower *D*_*50*_ are mixed with silty material from the artificial fill, and is therefore not considered representative for aeolian transported sediment.

In profile C, dune-building sediment was available at all sampling occasions, except for the first in October 2015 when it was finer, with *D*_*50*_ below 0.2 mm. In Profile A, sediment of appropriate grain size was only available on the last sampling occasion in October 2016. Profile B had only complete samples from three of the occasions and then showed availability in some of the sampling points along the profile, but not all.

### Model setup

The initial profile was schematized so that the total sand volume in the subaerial part of the profile was represented (Fig 10). Profile C, which consists of three dune rows, was schematized into one dune, while preserving dune volume and seaward dune crest position. This procedure is necessary as the CS-model can only simulate one row of dunes. The seaward dune slope angle, *β*_*S*_, was set equal to the angle of repose of sand (approximately 30–34 °), whereas the landward dune slope angle, *β*_*L*_, and maximum foreshore slope angle, *β*_*F*,*max*_, was obtained from the topographic observations.

**Fig 12:**
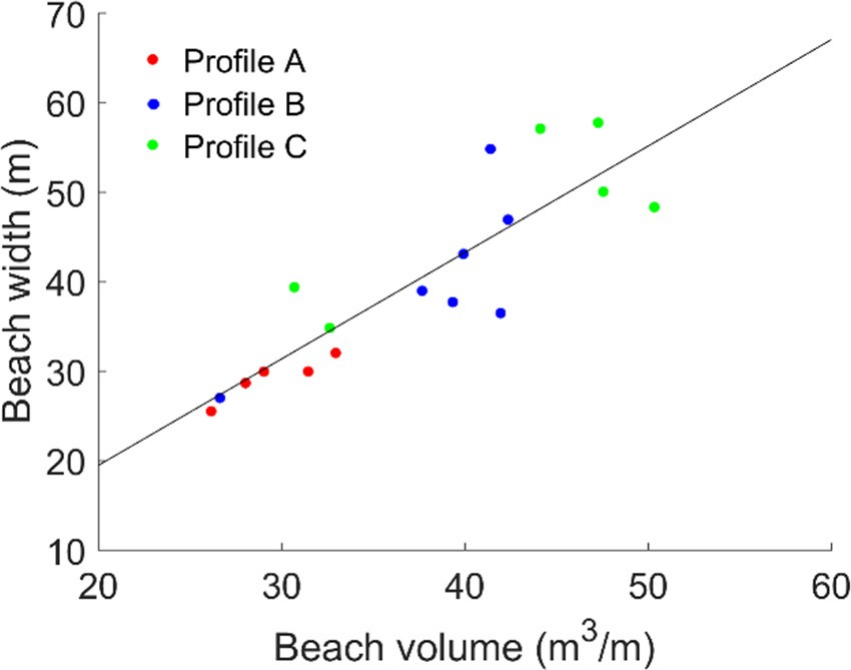
Observed relation between beach volume (V_berm_) and beach width (y_G_-y_S_).

The relationship between the beach width, *y*_*G*_-*y*_*S*_, and the beach volume, *V*_*berm*_, is determined from the topographic observations covering the profile from the dune foot (*y*_*S*_) to the intersection with MSL (*y*_*G*_) (Fig 12). A linear function was least-square fitted to the data yielding *y*_*G*_-*y*_*S*_ = 1.2*V*_*berm*_-4.3, with a coefficient of determination, *R*^*2*^, of 0.76.

The *D*_*50*_ used to calculate the fall velocity in the beach-bar exchange formulas [15] and employed in the aeolian transport equations was set to 0.2 mm for all three profiles. The dune foot height, *D*_*F*_, was estimated to 2 m for all profiles, based on an average of the topographic observations.

The depth of closure, *D*_*C*_, was calculated to be 5.7 m according to Hallermeier [63] and the width of the subaqueous part of the active profile was estimated to be 800 m (*B* = 800+*y*_*G*_-*y*_*S*_). The average sea temperature is assumed to be about 10°C [64].

The gradients in the longshore sediment transport rate was estimated to be 4.1 m^3^/m/year in profile A, 0 m^3^/m/year in profile B, and -3.0 m^3^/m/year in profile C, based on the vegetation line analysis [22]; thus, *q*_*LS*_ equals -4.1, 0, and 3 m^3^/m/year for profile A, B, and C respectively.

The simulation time step was set to one hour.

### Calibration and validation

The model was calibrated for the period 12/04/2010–21/04/2016 and then validated for the period 22/04/2016–17/01/2017. Literature values were used for all empirical coefficients [22] except for *C*_*S*_ and *C*_*W*_, which are the most important calibration parameters for the dune evolution.

Dune erosion was calibrated by adjusting the impact coefficient, *C*_*S*_, to fit the observed dune erosion. *C*_*S*_ describes the resistance of the dunes to wave erosion; the eroded volume from the dune during wave attack is proportional to *C*_*S*_ [15]. For profiles B and C, which involve dunes built up by aeolian transport with similar vegetation, *C*_*S*_ was calibrated to a common value of 7.5∙10^−5^. In profile A, the dune had initially aeolian transported sand, but was after April 2012 partly nourished with a silty material. The impact coefficient, *C*_*S*_, was here calibrated to a larger value of 5∙10^−4^, meaning that the dune is less resistant to erosion.

The empirical coefficient in the aeolian transport equation (Eq 7) *C*_*W*_ was calibrated to 1.0∙10^−5^, a common value for all profiles. Initial available sediment volume for aeolian transport, *V*_*w*_, was set to 0 m^3^/m in the calibration phase.

For this study, there was no information on the morphological evolution of the subaqueous part of the profile. The initial bar volume, which represents subaqueous deposits interacting with the beach deposits, was set to 20 m^3^/m for profile A, 70 m^3^/m for profile B, and 50 m^3^/m for profile C, to reproduce the evolution of the beach volume. For the validation, the bar volume at the end of the calibration period was used as the initial value.

**Fig 13:**
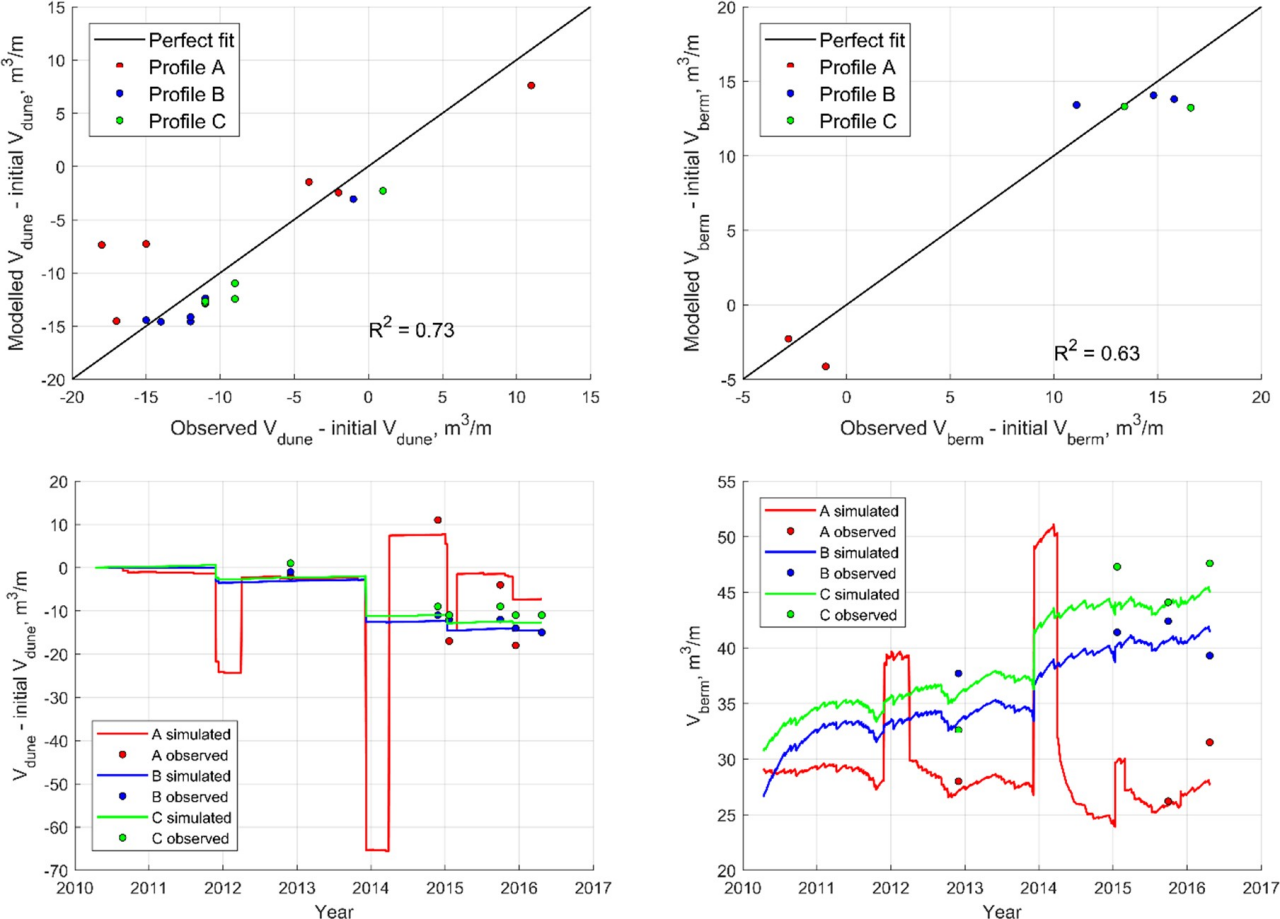
Dune and beach volume evolution for calibration period.

During the calibration period minor dune erosion events occur almost yearly and there are two major storm events with significant dune erosion in November 2011 and December 2013 (Fig 13). The sudden increases in dune volume in profile A are due to the dune nourishment episodes. In agreement with field observations, the aeolian transported volumes throughout the simulation period are small compared to the eroded volumes during these storm events. The dunes have not fully recovered at the end of the calibration period.

**Fig 14:**
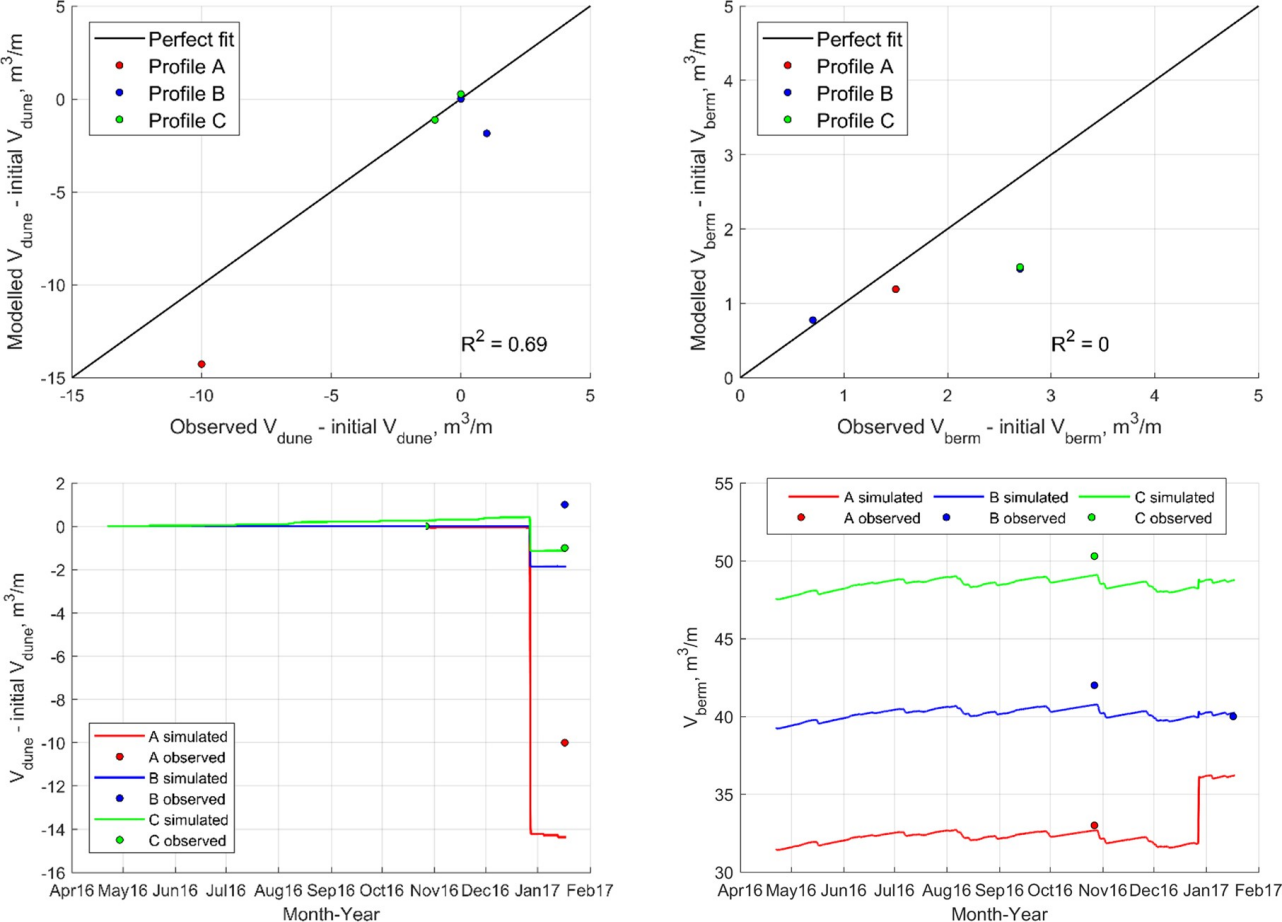
Dune and beach volume evolution for validation period.

The validation period contains one storm event in December 2016, with a clear signal of erosion in profile A and C (Fig 14). In profile B no erosion is observed, which is probably due to an error in the measured topographic data.

The performance of the calibration and validation is satisfactory. For the calibration period, the coefficient of determination, *R*^*2*^, equals 0.73 for the dune volume evolution and 0.63 for the berm volume evolution. For the validation period, *R*^*2*^ equals 0.69 for the dune evolution, but for the berm evolution, *R*^*2*^ equals 0 because of few observations with only small volume changes. The model sensitivity to the calibrated parameters was investigated by comparing a modified model to the calibrated model. (Table 2). The mean absolute deviation of dune and berm volume was calculated for a simulation period encompassing both the calibration and validation period. The sensitivity analysis showed that the model was most sensitive to changes in the duneha erosion impact coefficient, C_S_. Changes to the other parameters had only a minor impact on the dune and berm volume evolution.

**Table 2:** Result from sensitivity analysis of calibrated parameters.

Parameter	Modification	Mean absolute deviation from calibrated model (m^3^/m)	
		*V*_*dune*_	*V*_*berm*_
Initial V_W_	+10 m^3^	0.26	0.14
Initial bar volume, V_bar,1_	+25%	0.37	1.24
	-25%	0.39	1.22
C_W_	+25%	0.27	0.17
	-25%	0.27	0.19
C_S_	+25%	4.58	1.59
	-25%	4.28	1.66

However, there are uncertainties associated both with the topographic observations and the forcing. The beach profiles are derived from DEMs with resolution 1×1 m based on both LiDAR data and photogrammetry, and topographic surveys with GPS are carried out by both the local municipality and by researchers within this study. Therefore, it is uncertain whether some of the minor observed topographic developments are due to actual morphologic changes or artefacts related to different data acquisition methods. The eroded volumes under storms are on some occasions overestimated, and on other occasions underestimated. This can partly be due to both uncertainties in the topographic data and in the forcing.

In the dune erosion equation, the water level and wave properties both have large impact on the eroded volume. The lack of *in situ* water level gauge data introduces uncertainty when the water levels within the bay are corrected for the local wind setup. It is also possible that local variation in the wave climate occurs within the study area, which is not considered here; this could partly explain the large variation in the value of the impact coefficient, *C*_*S*_, between profile A and profiles B-C. Other possible explanations can be the mix of sediment in the dune in profile A, where the main part of the dune consists of sand with *D*_*50*_ around 0.2–0.3 mm and the front part consists of finer, silty material from the dune nourishment; and further, that vegetation is less abundant in profile A compared to B and C. The dune erosion formula used in the CS-model [55] was developed for sandy dunes with *D*_*50*_ ranging from 0.15 to 0.5 mm, and its validity outside this range of grain sizes is uncertain. Future coastal management approaches in the area are unknown; possibly the sediment characteristics in the dune may change. In the long-term simulations from 2018–2100, dune evolution in profile A is simulated with both the high and low calibrated value of *C*_*S*_.

**Fig 15:**
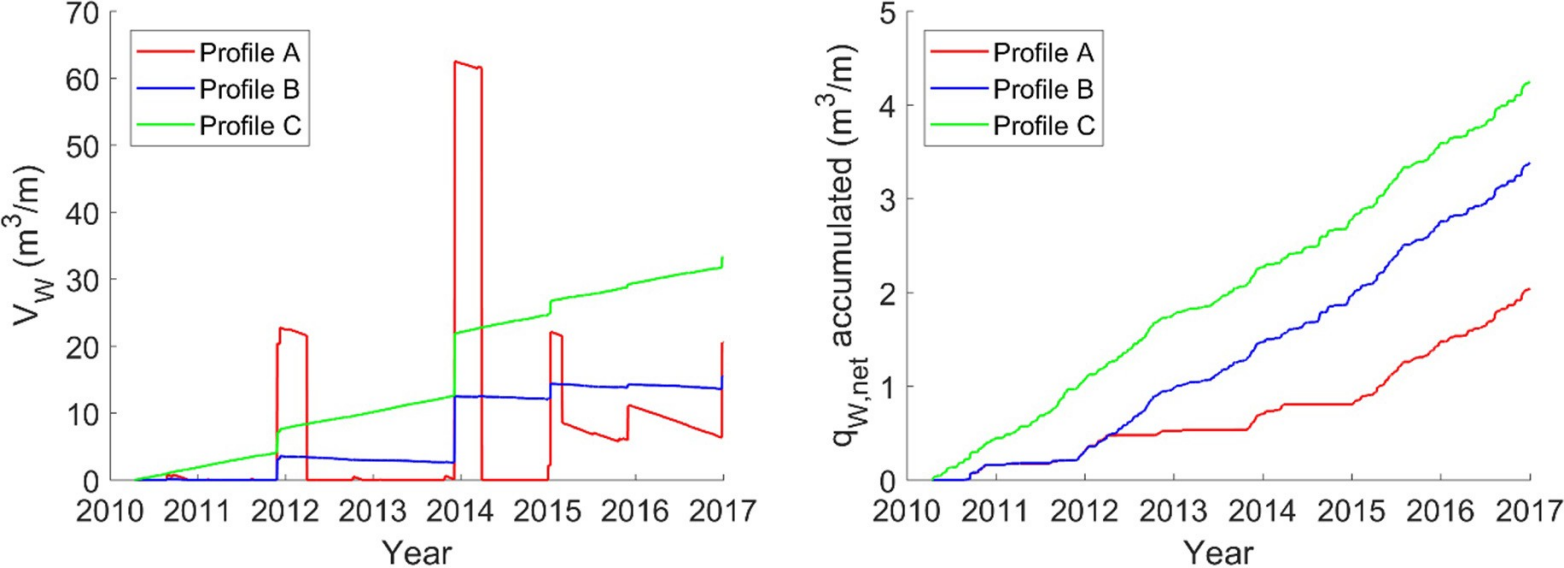
Available sediment for aeolian transport (V_W_) and accumulated aeolian transport (q_W_) during the calibration and validation periods.

The simulated yearly average aeolian transport amounts to 0.3 m^3^/m/year in profile A, 0.5 m^3^/m/year in profile B and 0.6 m^3^/m/year in profile C (Fig 15), which is significantly lower than in previous CS-model applications where a constant rate of about 10–11 m^3^/m/year was applied in all three profiles [22]. The new results are more realistic when compared to the observed long-term evolution. In the accreting profile C, the vegetation line has advanced 0.3 m/year since the 1940s [22], which is approximately equal to an accumulation of 0.6 m^3^/year considering the average dune height of 2 m.

The simulation results, with the highest aeolian transport in profile C and lower transport in profile A, are in line with the topographic observations. The simulated differences in the average annual transport rates are due to variations in sediment supply between the different profiles as well as the variation in beach width. The four sets of sediment samples taken from October 2015 to October 2016 display variations both in space and time (Fig 11), where profile C has the most available sediment, profile B less, and profile A only intermittent availability during the period 2010–2016. This pattern is reflected in the simulated available sediment (Fig 15). However, the simulation results show that there should be sediment available for aeolian transport within all three profiles during the actual sampling period, which was not supported by the sediment grain size data. An explanation for this could be that the material eroded from the dune in profile A before the sampling period was finer than the appropriate grain size for dune build up, due to the silty nourishment, and thus should not have been accounted for in the available sediment volume, *V*_*W*_.

### Long-term simulation with sea level rise

Long-term beach and dune evolution from 2018–2100 was simulated with a range of SLR scenarios from IPCC [65]. The simulations were run with 10 sets of 83 years of input data, obtained by randomly shuffling years (July–June) from 01/07/1976–30/06/2016 with simultaneous values of wind, waves, and water levels relative to MSL. The three different scenarios of SLR until 2100 were defined as mean low 0.44 m, mean high 0.74 m, and likely upper range high 0.98 m, relative to the average MSL during the years 1986–2005. The first scenario corresponds to the RCP 2.6 scenario and the latter two to the RCP 8.5 scenario in the fifth assessment report by IPCC [65]. The SLR rate was derived from fitting quadratic polynomials to the data provided by IPCC (Table AII.7.7 in [66]). A post glacial uplift of 1.3 mm/year [67] was subtracted from the computed SLR rates and the scenarios were compared to a baseline scenario with no relative SLR, *i*.*e*. where SLR compensates exactly the post-glacial uplift.

The regional climate change prognosis does not predict any changes to the wind climate [67]; therefore no changes in wind speed or direction were considered in this study. The same holds for the wave climate, which is dominated by regionally wind-generated waves.

Profile A was simulated with two different values on the impact coefficient, *C*_*S*_; the calibrated value of 5∙10^−4^, which is assumed to partly depend on the dune nourishments during the calibration period, and the lower value of 7.5∙10^−5^, which was estimated for profiles B and C.

**Fig 16:**
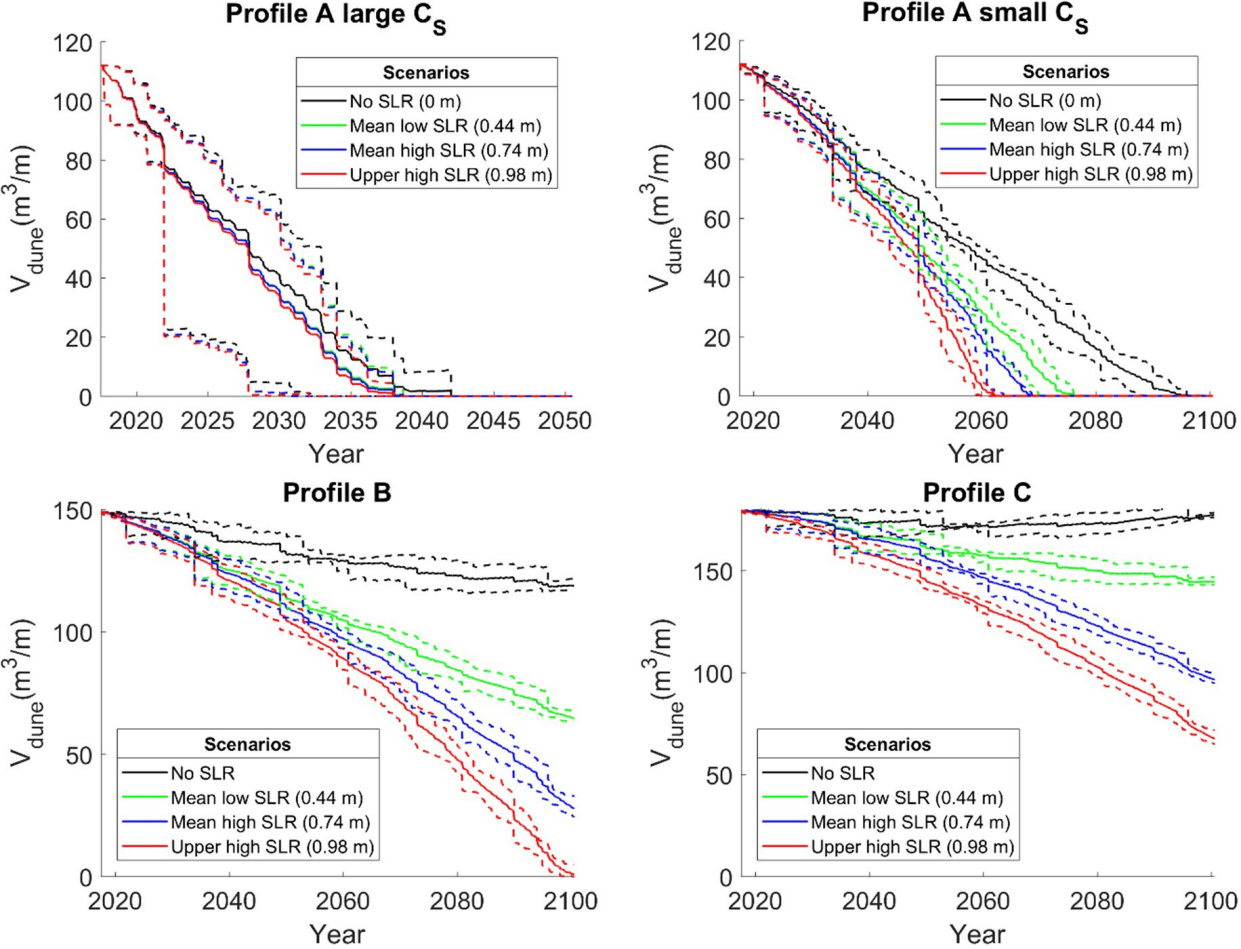
Simulated dune volume evolution. Solid lines represent mean value, and the dashed lines represent min and max values from the 10 simulations for each scenario.

In all profiles, dune volumes decrease for all three scenarios, including profile C despite input of sediment due to a negative gradient in longshore transport (Fig 16). In Profile A, the dune erodes away completely before year 2100, both with the large and small dune erosion coefficient, *C*_*S*_. In the simulations with large *C*_*S*_, dune erosion is more important than the effect of SLR, also without SLR the dune is eroded away quickly. Taking into account all SLR scenarios, the dunes are eroded away completely within the period 2050–2075 with the lower *C*_*S*_ coefficient, compared to 2027–2037 with the higher *C*_*S*_ coefficient. This evolution is explained by the long-term trend of beach erosion and the severe storms in the last decade that are present in the data set.

For profile B, the dune only disappears completely in the upper high SLR scenario, but the volume is markedly diminished also in the other two scenarios. In Profile C, the effect of SLR is compensated by the negative gradient in the longshore transport, which is added as a constant transport rate to the beach volume. Still, the long-term simulation without SLR does not show a net dune growth, which could be expected on an accreting beach. This can be explained by the severe storm events in 2011 and 2013 that are present in the data set.

**Fig 17:**
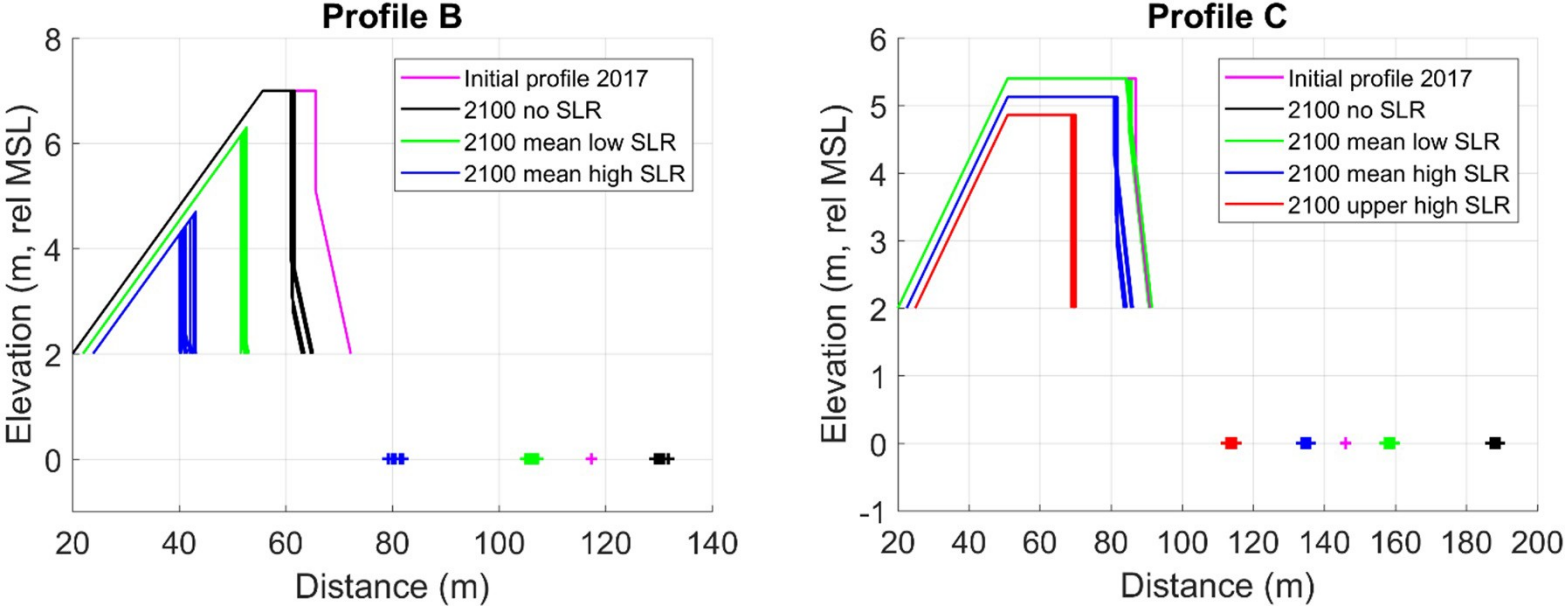
Simulated beach–dune evolution for profile B and C. The ‘+’ indicates intersection with MSL.

The long-term observation of dune evolution based on aerial photos from the 1940s contains a period during 1960s and 1970s that was unusually calm with respect to storminess [68]. If the wind climate from 1976–2016 is representative for future conditions, model results indicate that the aeolian transport capacity will not be sufficient to repair storm erosion. In profiles B and C, the available sediment for aeolian transport accumulates faster than the aeolian transport rate, the gradient in longshore transport cause the beach volume to increase and the shoreline to extend seawards (Fig 17). The wider beach increases frictional losses, so that dune erosion decreases which explains the trend change in dune volume evolution in profile C for the low and baseline scenarios (Fig 16). The dune erosion and recovery during the simulation period are subtracted from, or added to, the foredune. In reality, new foredune ridges could form during periods of recovery, however, this is not possible with the present simplified dune schematization of the CS-model.

The dunes are also losing sediment due to the change of reference dune foot elevation when the sea level is rising, causing the dune crest height to decrease (Fig 17). In profile B the horizontal dune crest erodes away completely and the dune attains a triangular shape, so that the dune crest is further lowered due to storm erosion. None of the simulations of profile B and C results in landward dune translation. In profile B, with no gradient in the longshore transport, SLR changes the sediment budget from stable to eroding for all scenarios. Profile C is still accreting in the low scenario but changes into a negative sediment budget in the mean high and upper high scenarios. In the scenarios with negative sediment budget, the aeolian transport capacity is too low to restore the dune ramp, which is supposed to produce landward translation between consecutive erosion events.

## Discussion and concluding remarks

In this study, the CS-model was expanded to simulate beach and dune evolution at decadal to centennial time scales under SLR. Model development was based on morphological concepts from the literature, which were translated into mathematical formulations that were numerically solved. The proposed model was applied to a data set from Ängelholm Beach, Sweden. First, the model was calibrated and validated for a seven-year period, and then the beach and dune evolution was simulated from 2018–2100 for a range of SLR scenarios.

Topography and grain size data suggests that the morphological evolution at Ängelholm Beach conform to the concepts that were introduced in the CS-model. The different profile characteristics fit the conceptual sediment budget model by Psuty [24] where the eroding and stable dunes in profiles A and B are the highest and the accumulating profile C displays a lower, prograding dune-ridge topography. The differences in distribution of *D*_*50*_ over the profiles are in line with the hypothesis that available sediment is a controlling factor for aeolian transport and that preconditions for aeolian transport are variable both in space and time. However, although there was sediment available, the aeolian transport that was observed in the stable and accreting profiles B and C were not of sufficient rates to repair the storm erosion during the study period.

Overall, the results of the calibration and validation against field data were satisfactory and the long-term predictions of beach and dune evolution under different SLR scenarios showed reasonable results. The CS-model illustrated the relative importance of longshore transport gradients, storm erosion, aeolian transport, and SLR for the profile evolution. The simulations demonstrated the importance of extreme storm events for decadal-scale dune evolution, and confirmed the need of integration of physical processes acting on multiple time scales [21].

At Ängelholm Beach the aeolian transport capacity, rather than sediment availability, limited the dune growth in the long-term simulations. In the case with a positive sediment budget (the low SLR scenario in profile C), the available sediment was accumulating faster than the aeolian transport capacity, so that the shoreline extended seawards, widening the beach. For the profiles with negative sediment budget, the dune ramp did not become fully developed until the sediment available for aeolian transport had eroded away, suggesting that aeolian transport capacity in relation to dune erosion rates is too low to result in landward dune migration. The model assumes that the dune ramp is built entirely by wind-blown sand. Field studies suggest that the main part of the ramp is built from wind-blown sand [36], however, in future studies it would be worthwhile exploring if an avalanche mechanism should be included in the model to facilitate the development of dune ramps.

Precipitation is not included in the model, but may be an important limiting factor [35]. At beaches with seasonal ice and snow cover the effect on aeolian transport may be significant, especially if transport is inhibited during periods with strong winds [36]. In areas with significant impact of rain, snow, and ice, the model could be improved through modifying sediment availability during wet or frozen conditions to restrict aeolian transport. Blowouts may also be an important factor for landward foredune migration [40] but are difficult to include in a 1-D model.

The CS-model assumes an equilibrium profile and therefore the Bruun rule fits well with the model concept. Introducing a more realistic profile representation to the CS-model would introduce a higher degree of model complexity, which is not desired. It is however important to keep the limitations of the equilibrium concept in mind when applying the CS-model and the Bruun rule. Furthermore, the depth of closure is time scale dependent. Over longer periods, the probability of storm events mobilizing sediment at deeper water increases [69]. Therefore, the depth of closure adopted in the CS-model should be chosen with consideration to the length of the simulation period.

In conclusion, the modified CS-model, including SLR related processes and a more advanced transport and dune evolution scheme, provides fast and robust simulations with reasonable results. The simplified model approach enables robust, long-term simulations and makes the model suitable for probabilistic simulations. The results presented in this study are promising, but more studies with longer-term data sets are required to validate the proposed numerical formulations more thoroughly.

## List of symbols

*A*_*b*_ Coefficient describing fraction of nourishments available for aeolian transport [–]
*A*_*e*_ Coefficient describing fraction of sediment deposited on dune crest in a negative sediment budget [–]
*A*_*q*_ Coefficient describing fraction of longshore and ‘Bruun Rule’ transport available for aeolian transport [–]
*A*_*s*_ Coefficient describing fraction of sediment deposited on dune crest in a stable sediment budget [–]
*A*_*W*_ Coefficient in critical shear velocity equation [–]
*B* Width of active profile [m]
*B*_*Bruun*_ Width of active profile in the original Bruun rule [m]
*B*_*dry*_ Dry beach width [m]
*c*_*f*_ Empirical friction coefficient for wave runup [–]
*C*_*S*_ Dune erosion impact coefficient [–]
*C*_*W*_ Empirical coefficient in aeolian equilibrium transport formula [–]
*d* Grain size [mm]
*D*_*50*_ Median grain size [m]
*D*_*50*_^*ref*^ Median reference grain size [m]
*D*_*C*_ Depth of closure [m]
*D*_*F*_ Dune foot height [m]
*F* Fetch (aeolian transport) [m]
*g* Standard acceleration due to gravity [m/s^2^]
*h* Height of active profile [m]
*H*_*m0*_ Energy based significant wave height [m]
*H*_*rms*_ Deep water root-mean-square wave height [m]
*i* Index, time step [–]
*K*_*W*_ Empirical coefficient in aeolian equilibrium transport formula [–]
*L*_*0*_ Mean deep water wave length [m]
*MSL* Mean sea level [m]
*m*_*WE*_ Potential aeolian transport rate [kg/s/m]
*n* Number of timesteps [–]
*P* Porosity [–]
*q*_*D*_ Transport rate of eroded sediment from the dune [m^3^/s/m]
*q*_*L*_ Transport rate of eroded sediment from the dune front to the landward side of the dune [m^3^/s/m]
*q*_*LS*_ Transport rate due to gradients in longshore transport [m^3^/s/m]
*q*_*S*_ Transport rate of eroded sediment from the dune to the beach [m^3^/s/m]
*q*_*SLR*_ Transport rate of sediment to compensate for sea level rise, ‘Bruun Rule’ transport [m^3^/s/m]
*q*_*W*_ Onshore component of aeolian transport [m^3^/s/m]
*q*_*WE*_ Volumetric aeolian transport rate [m^3^/s/m]
*q*_*WF*_ Potential aeolian transport rate corrected for fetch length [m^3^/s/m]
*R* Runup [m]
*R'* Runup corrected for friction over beach [m]
*R*^*2*^ Coefficient of determination [–]
*R*_*Bruun*_ Shoreline retreat due to sea level rise [m]
*S* Dune height [m]
*S*_*max*_ Maximum dune height limiting vertical dune growth for positive sediment budget [m]
*S*_*new*_ Dune height after *SLR* [m]
*S*_*old*_ Dune height before *SLR* [m]
*S*_*SLR*_ Height of sea level rise [m]
*SLR* Sea level rise [–]
*SWL* Still water level [m]
*t* Time step [–]
*T*_*bud*_ Significant time scale for sediment budget [s]
u* Shear velocity [m/s]
u*_c_ Critical shear velocity [m/s]
*u*_*z*_ Wind speed at *z* m elevation [m/s]
*u*_*z*,*c*_ Critical wind speed at *z* m elevation [m/s]
*V*_*bar*_ Volume of subaqueous deposits [m^3^/m]
*V*_*berm*_ Volume of beach above mean sea level, between shoreline and dune foot [m^3^/m]
*V*_*beach*_ Total beach volume [m^3^/m]
*V*_*beach*, *new*_ Total beach volume after *SLR* [m^3^/m]
*V*_*beach*, *old*_ Total beach volume before SLR [m^3^/m]
*V*_*dune*_ Dune volume [m^3^/m]
*V*_*nour*_ Nourishment volume [m^3^/m]
*V*_*ramp*_ Dune ramp volume [m^3^/m]
*V*_*SLR*_ Eroded volume from Vbeach, due to *SLR* [m^3^/m]
*V*_*w*_ Volume of sediment available for aeolian transport [m^3^/m]
*x* Horizontal travel distance of wave front [m]
*y’*_*L*_ Landward dune crest length coordinate [m]
*y’*_*S*_ Seaward dune crest length coordinate [m]
*y*_*G*_ Shoreline length coordinate [m]
*y*_*L*_ Landward dune foot length coordinate [m]
*y*_*R*_ Runup limit length coordinate [m]
*y*_*S*_ Seaward dune foot length coordinate [m]
*z* Wind gauge elevation [m]
*z*_*0*_ Roughness height [m]
*β*_*F*_ Average beach slope angle [°]
*β*_*F*,*max*_ Maximum foreshore slope angle [°]
*β*_*L*_ Landward dune slope angle [°]
*β*_*S*_ Seaward dune slope angle [°]
*δ* Empirical coefficient to account for fetch effect [m^-1^]
Δ*t* Length of time step [s]
Δ*V*_*T*_ Change of volume in beach- dune system to determine sediment budget [m^3^/m]
*κ* von Karman’s constant [–]
*Θ* Wind angle against shore normal [°]
*ρ*_*a*_ Air density [kg/m^3^]
*ρ*_*s*_ Sand density [kg/m^3^]
